# Harnessing Raman spectroscopy and multimodal imaging of cartilage for osteoarthritis diagnosis

**DOI:** 10.1038/s41598-024-83155-3

**Published:** 2024-12-28

**Authors:** Anna Crisford, Hiroki Cook, Konstantinos Bourdakos, Seshasailam Venkateswaran, Douglas Dunlop, Richard O. C. Oreffo, Sumeet Mahajan

**Affiliations:** 1 School of Chemistry, Faculty of Engineering and Physical Sciences, University of Southampton, Life Sciences Building 85, University Road, Highfield, Southampton, SO17 1BJ UK; 2 Human Development and Health, Faculty of Medicine, University of Southampton, Southampton, UK; 3 Institute for Life Sciences, University of Southampton, Southampton, UK; 4 Precision Healthcare University Research Institute, Queen Mary University of London, London, UK

**Keywords:** osteoarthritis diagnosis, cartilage layers, Fingerprint Raman, Multimodal imaging, Deep tissue imaging, Chemical biology, Biomarkers, Diseases, Medical research, Chemistry

## Abstract

Osteoarthritis (OA) is a complex disease of cartilage characterised by joint pain, functional limitation, and reduced quality of life with affected joint movement leading to pain and limited mobility. Current methods to diagnose OA are predominantly limited to X-ray, MRI and invasive joint fluid analysis, all of which lack chemical or molecular specificity and are limited to detection of the disease at later stages. A rapid minimally invasive and non-destructive approach to disease diagnosis is a critical unmet need. Label-free techniques such as Raman Spectroscopy (RS), Coherent anti-Stokes Raman scattering (CARS), Second Harmonic Generation (SHG) and Two Photon Fluorescence (TPF) are increasingly being used to characterise cartilage tissue. However, current studies are based on whole tissue analysis and do not consider the different and structurally distinct layers in cartilage. In this work, we use Raman spectroscopy to obtain signatures from the superficial (top) and deep (bottom) layer of healthy and osteoarthritic cartilage samples from 64 patients (19 control and 45 OA). Spectra were acquired both in the ‘fingerprint’ region from 700 to 1720 cm^− 1^ and high-frequency stretching region from 2500 to 3300 cm^− 1^. Principal component and linear discriminant analysis was used to identify the peaks that contributed significantly to classification accuracy of the different samples. The most pronounced differences were observed at the proline (855 cm^− 1^ and 921 cm^− 1^) and hydroxyproline (877 cm^− 1^ and 938 cm^− 1^), sulphated glycosaminoglycan (sGAG) (1064 cm^− 1^ and 1380 cm^− 1^) frequencies for both control and OA as well as the 1245 cm^− 1^ and 1272 cm^− 1^, 1320 cm^− 1^ and 1345 cm^− 1^, 1451 cm^− 1^ collagen modes were altered in OA samples, consistent with expected collagen structural changes. Classification accuracy based on Raman fingerprint spectral analysis of superficial and deep layer cartilage for controls was found to be 97% and 93% on using individual/all spectra and, 100% and 95% on using mean spectra per patient, respectively. OA diseased cartilage was classified with an accuracy of 88% and 84% for individual/all spectra, and 96% and 95% for mean spectra per patient based on analysis of the superficial and the deep layers, respectively. Raman spectra from the C-H stretching region (2500–3300 cm^− 1^) resulted in high classification accuracy for identification of different layers and OA diseased cartilage but low accuracy for controls. Differential changes in superficial and deep layer cartilage signatures were observed with age (under 60 and over 60 years), in contrast, less significant differences were observed with gender. Prominent chemical changes in the different layers of cartilage were preliminarily imaged using CARS, SHG and TPF. Cell clustering was observed in OA together with differences in pericellular matrix and collagen structure in the superficial and the deep layers correlating with the Raman spectral analysis. The current study demonstrates the potential of Raman Spectroscopy and multimodal imaging to interrogate cartilage tissue and provides insight into the chemical and structural composition of its different layers with significant implications for OA diagnosis for an increasing aging demographic.

**Supplementary Information:**

The online version contains supplementary material available at 10.1038/s41598-024-83155-3.

## Introduction

Musculoskeletal disorders are the second most common cause of disability worldwide. Osteoarthritis (OA), is the most common form of arthritis, resulting in an estimated 8.75 million of the UK population over 45 years of age seeking treatment annually; and a global prevalence of over 500 million^1^. Indeed, the World Health Organization reported that OA is the single most common cause of disability in the older population^2^. The health burden of osteoarthritis will continue to rise with increasing obesity, sedentary lifestyle and an increasing growing population of 60 years or older, which is expected to double by 2050^2^. OA is a late-onset, complex disease of the joint that is typically characterised by degeneration and thinning of the articular cartilage, changes in synovium and subchondral bone causing loss in lubrication in a joint^3^.

OA affects the whole joint; however, the main changes occur in articular cartilage leading to the complete loss of articular cartilage in severe cases. Articular cartilage is a connective tissue around 2 to 4 mm deep, present in joints of knees, hips, spine and fingers which serves as a shock absorber facilitating transmission of loads and smooth painless movements of joints. Articular cartilage is avascular, aneural and a lymphatic supply is nourished by the synovial fluids. Articular cartilage is made up of a dense extracellular matrix that predominantly comprises water and electrolytes (60–85% by weight) as well as collagen, non-collagenous proteins, proteoglycans, lipids, phospholipids and a sparse distribution of highly specialized cells (chondrocytes, 10% by weight) that produce the cartilage matrix^4–7^. The dry weight of articular cartilage is primarily collagen (60%), however, the protein composition of cartilage is complex and varies depending on the depth of the tissue and distance from the chondrocytes^8–10^.

Morphologically, cartilage can be divided into 3 layers: superficial, middle and deep (part of which is calcified, separated from the bone with a tidemark) zones and the key characteristics of each of these layers are summarized in Table 1^10–14^. While chondrocytes are found in all three layers, their shape, orientation and expression of collagen type as well as other matrix proteins vary depending on the layer of cartilage in which they are present^10,15,16^. Articular cartilage comprised of predominantly type II collagen (up to 90%) but it also contains types IX, XI and VI^16–18^. Type I and II collagens are fibrillar and have high activity in second harmonic generation^19^.

**Table 1:** Collagen phenotype across cartilage layers.

Cartilage layer	% of the cartilage volume	Morphology of chondrocytes	Main collagen constituents
Superficial	10–20%	Elongated/flattened	Mainly Type II (also, IX and XI) condensed collagen fibers running parallel to the joint
Middle	40–60%	Round	Mainly Type II collagen fibers that are randomly arranged and slightly tilted. Transitioning from a more parallel (near the superficial zone) to a more perpendicular orientation (towards the deep layer).
Deep	~ 30%	Round, arranged in columns	Type II collagen fibers running perpendicular to the joint and across the tidemark.

Collagen type VI is found almost exclusively in the pericellular matrix (PCM), the area surrounding the chondron within which the chondrocytes are located providing structural integrity and facilitating communication with the extracellular matrix^17,20^. The PCM contains biglycan and decorin proteins which connect type VI collagen fibers with type II collagen fibers, providing stable matrix in the immediate proximity of chondrocytes^20^.

At the onset of OA, two important changes are known to occur within the cartilage: (i) chondrocytes undergo proliferation, cell death, autophagy and form clusters^15,16,21^ and, (ii) the fluid in the cavity of the joints, the synovial fluid, becomes rich in inflammatory cytokines, complement components and plasma proteins^22,23^. The primary inflammatory mediators have been identified as lipid molecules, prostaglandins and leukotriene^24,25^ and other molecules released by white adipose tissue^26^. Cartilage matrix degradation starts from the superficial layer and progresses to the deeper cartilage layers^12,27,28^. However, the aetiology and molecular mechanisms responsible for the onset and progression of osteoarthritis remain poorly understood and their potential for diagnostic prediction has not been fully explored^29,30^. The current gold-standard techniques for diagnosis of osteoarthritis focus on observation of the changes in morphology and bulk structure of the tissue. Techniques employed include advanced radiography, four-dimensional CT scan, CT arthrography, nuclear medicine techniques such as SPECT/CT, PET/CT, PET/MRI, three-dimensional quantitative cartilage morphometry as well as MRI and x-rays^3,29,31^. These imaging techniques detect morphological changes such as narrowing of the space margin between the two bones in the joint as a consequence of cartilage loss or accumulation of synovial fluids and hence are typically limited to advanced stages of osteoarthritis, following disease progression^27^.

Early diagnosis of OA is highly desirable to enable timely implementation of lifestyle changes and medical interventions to reduce pain, improve mobility and patient quality of life. There is currently no cure for osteoarthritis and treatment regimens are targeted at alleviating inflammatory symptoms or arthroplasty such as a prosthetic joint replacement. Efficacious application of minimally invasive and non-destructive techniques such as Raman spectroscopy that can directly (and potentially in vivo) detect subtle biochemical changes that occur within the cartilage at onset and during osteoarthritis and, critically will provide markers for diagnosis of osteoarthritis before symptoms appear and thus aid the discovery of new pharmacological interventions to halt OA progression, remains a key research goal. Raman spectroscopy is highly applicable for in vivo diagnosis as well as for evaluation of ex vivo articular cartilage samples due to its insensitivity to water and hence, can provide information on constituent molecules as well as their interactions with surrounding molecules despite its high water content. Several characteristic Raman bands have been assigned to vibrational modes of constituent molecules in cartilage tissue. These include modes assigned to C-O stretching; amide I, random coil (1668 cm^− 1^), Amide I, collagen secondary structure (1640 cm^− 1^), C = C stretching; phenylalanine, tryptophan (1606 cm^− 1^), Amide II (1557 cm^− 1^), CH_2_/CH_3_ scissoring; collagen and other proteins (1450 cm^− 1^), COO^−^; GAGs (1424 cm^− 1^), CH_3_; GAGs (1380 (proteoglycan) cm^− 1^), (NH_2_) bending; amide III, α-helix (1270 cm^− 1^), Amide III, α-helix (1260 cm^− 1^), (NH_2_) bending; amide III, random coil (1245 cm^− 1^), Amide III, random coil (1235 cm^− 1^), Pyranose ring (1163 cm^− 1^, 1042 cm^− 1^), C-C, C-OH, C-N stretching, C-O-C glycosidic linkage (1125 cm^− 1^), SO_3_^−^ stretching; GAGs (chondroitin sulfate) (1063 cm^− 1^), Phenylalanine ring breathing (1003 cm^− 1^), C-C stretching; collagen, α-helix (941 cm^− 1^), C-C stretching; hydroxyproline (875 cm^− 1^), C-C stretching; proline (858 cm^− 1^) and C-C stretching; protein backbone (816 cm^− 1^)^32–43^.

For the diagnosis of osteoarthritis, it is important to identify the spectral changes in cartilage, that is, the changes in the peak patterns instead of intensity of individual peaks. This is carried out through multivariate analysis such as principal component and linear discriminant analysis (PCA and LDA) wherein the spectral loadings link the variance of the peaks across classes to the original spectra. Changes in the ratio of the intensity of certain vibrational modes also provide valuable insight and can potentially provide diagnostic information. The current study has applied PCA and LDA to understand the spectral differences between the different layers in healthy and OA cartilage for their unsupervised and supervised classification accuracy.

Raman Spectroscopy has been used by a number of groups to study human osteoarthritic cartilage^34,36,38–41,44^. Takahashi et al. completed a preliminary study on 5 arthritic human tibial cartilage samples retrieved from knee arthroplasty and found an increase in the relative intensity ratio between the Raman bands of collagen located at 1241 and 1269 cm^− 1^ (amide III doublet) with increasing degradation grades indicating diagnostic potential^39^. A recent feasibility study on 47 patient samples extended the aforementioned work and observed a decrease in sulfated glycosaminoglycans and proteoglycans and increase in collagen disorganization with severity of hip osteoarthritis^40^. Additionally, Raman spectroscopy was used to predict severity of OA in human knees cartilage by studying changes in spectral bands from synovial fluid, associated with secondary structure of proteins^45^. Severity of the natural degradation of cartilage in disease, also, physically and chemically induced cartilage damage were successfully assessed by multivariant analysis of Raman spectra^46^. Similarly the diagnostic potential of Raman spectroscopy to study alterations in collagen structure in disease diagnosis was explored by multiple groups and reviewed by Martinez et al.^36^ but the different layers of cartilage were not taken into account. These studies confirm the diagnostic potential of Raman spectroscopy, however, to date, there has been no published evidence of the changes in the different layers of cartilage characterized by Raman spectroscopy or, the use of the differential signatures between the cartilage layers for osteoarthritis diagnosis in patients.

The current study has harnessed Raman Spectroscopy to characterize osteoarthritic and non-osteoarthritic patient samples of cartilage derived from femoral heads post hip arthroplasty. We investigated changes in the different layers of cartilage to identify discrete differences in their molecular composition. PCA and PC loadings were applied to understand the contribution of different vibrational modes to unsupervised classification accuracy. SVM was used for supervised classification accuracy. The dependence of the Raman spectral signatures from the different layers of cartilage with gender as well as age was examined. We further used the multimodal imaging techniques of coherent anti-Stokes Raman scattering (CARS), second harmonic generation (SHG) and two-photon autofluorescence (TPF) microscopy on representative samples to correlate any changes in the structure and organization of different components in the superficial and deep layers with changes observed by Raman spectral analysis. The current work aims explored the potential of OA diagnosis using the Raman signatures of different cartilage layers offering significant diagnostic implications for an increasing aging demographic.

## Materials and methods

### Sample preparation

Femoral head specimens were collected from patients undergoing total hip arthroplasty at Southampton General Hospital (SGH) and Spire Southampton Hospital. All donors provided informed written consent before obtaining samples. The study protocol was approved by the University of Southampton’s local Ethics and Research Governance Office (ERGO 71875) and by the National Health Authority – North West – Greater Manchester East Research Ethics Committee (18/NW/0231) and conformed to the ethical guidelines of the Helsinki Declaration. All work undertaken in this study was performed in accordance with the relevant guidelines and regulations approved by the University of Southampton and by the National Health Authority. All femoral heads were clinically evaluated and classified as either osteoarthritic (Mankin score 3 to 4) or non-osteoarthritic.

**Fig. 1:**
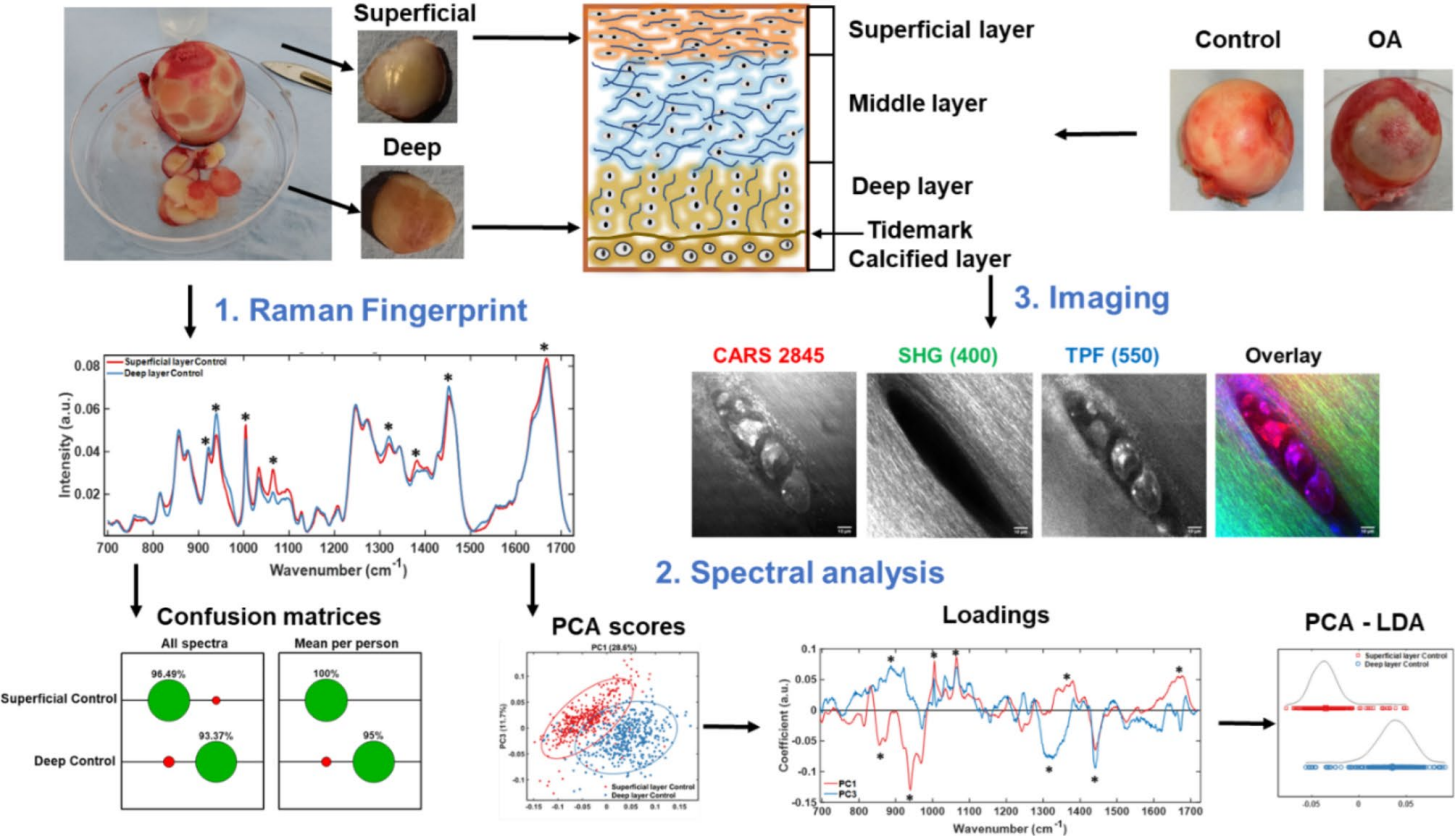
Workflow and methodology used in this work illustrated using control cartilage samples. Top right shows femoral heads received post-surgery and the samples generated for analysis of superficial and deep layers (top left). The schematic shows the layered structure of the cartilage. (1) Shows the mean Raman spectra for the different cartilage layers (2) Spectra were analysed using Principal Component Analysis (PCA) and the scatter plot and loadings are shown. PCA was cascaded into Linear Discriminant Analysis (LDA) which improves the separation between classes. Confusion matrices show the classification accuracy into superficial and deep layers based on its spectral signatures from all individual spectra to investigate heterogeneity and average spectra per patient for diagnostics. Confusion matrices were created with SVM analysis of the spectra directly. (3) Representative multimodal images of articular cartilage showing lipids and cell phospholipid bilayers (CARS), collagen fibres (SHG) and autofluorescent biological molecules (TPF). In overlay, the three modalities are combined: CARS (red), SHG (green), TPF (blue). Scale bar 10 μm.

All osteoarthritic donors (*n* = 45, 24 female and 21 male) had no signs of osteoporosis or any other degenerative diseases. All non-osteoarthritic donors (*n* = 19, 10 female and 9 male) had osteoporosis but no obvious detectable osteoarthritis or other cartilage degenerative disease and, hence, were treated as ‘healthy’ controls as confirmed by a consultant orthopaedic surgeon (Professor Douglas Dunlop) (Table 2). Cartilage slices were taken from areas with close to full thickness, corresponding to low disease severity (early-OA) and non-weight bearing areas, which would undergo increased thinning, under aseptic conditions, using a scalpel blade able to cut up to the subchondral bone (Fig. 1), washed (PBS, 1X), fixed (4% formaldehyde, 4 °C, 72 h, gentle shaking), washed (PBS, 3X) and stored in PBS at 4 °C in a refrigerator until Raman spectra were obtained.

**Table 2:** Patient age and gender used in the current study.

Demographics	*N*	Mean ± SEM
Female control	10	71.3 ± 5.94
Male control	9	72 ± 5.07
Female OA	24	68.9 ± 2.76
Male OA	21	69.6 ± 2.31

### Raman spectroscopy

Fixed cartilage slices (4 per patient) were chosen randomly, washed (distilled water, X2) and Raman spectra (fingerprint region including 615–1722 cm^− 1^ and the CH_2_ stretching region from 2496 to 3265 cm^− 1^) obtained using a pre-calibrated (to 520 cm^− 1^ reference peak of silicon) Renishaw InVia Raman microscope (785 nm laser, 10 mW, x50 (0.75 NA) short working distance (~ 200 μm) objective, exposure 5 s, 3 accumulations) controlled by the proprietary Renishaw WIRE4.1 software that enabled cosmic ray removal. Whilst control cartilage mostly gave high signal-to-noise ratio (SNR) some of the samples from OA cartilage, did not give such robust results with a single spectral accumulation and hence, we carried out 3 accumulations for each spectrum to have consistency of acquisition conditions across all samples. The spectral measurements from tissue samples were collected in the back-scattering (episcopic) configuration to be close to a potentially translatable configuration; laser penetration was around 100 μm into the tissue and thus data was collected from the surface of the cartilage. Additionally, this study only established “signatures” for (top) Superficial and (bottom) Deep layer of cartilage. The superficial and deep layers were easily and consistently identifiable on the cartilage slices; with the superficial surface appearing smooth and “shiny”, and deep surface appearing darker and “rough”. Another corroborating spectral marker was the height of the 1065 cm^−1^ peak in the unprocessed Raman spectra, which appeared consistently small or close to non-existent in the deep layer.

Each piece of cartilage was around 1–2 cm long and we approximately acquired spectra every 500 microns. The thickness was variable but was typically from 1 to 3 mm thick. Thus, we covered only a small portion of the cartilage on the femoral head but believe that it gave us representative data. Late stage OA femoral heads often did not contain much cartilage left and satisfied this method.

Hence, while the cartilage on the femoral heads is the thickest, compared to other joints, the thickness is not important for our Raman spectroscopy measures as it is not expected to affect the data, at least when the cartilage thickness ranged from 1 to 3 mm as described above.

As the cartilage samples are not completely flat, especially from OA patients, excess spectra than necessary were taken to ensure increased SNR, as it is not evident while doing recordings if they were of the good quality (SNR) due to the high background, possibly due to collagen autofluorescence signal.

Four cartilage slices per patient were chosen at random for unbiased indicative coverage of the cartilage across the femoral head. Raman measurements were from first superficial and then the deep layer of each slice, kept moist throughout with addition of dH_2_O around the sample. At least 5 repeats were made from points across the cartilage surface at intervals of the order of 100’s µm, for ≥ 20 spectra from each layer (superficial and deep) per patient.

Raman data and analysis from CH_2_ stretching region (2496–3265 cm^− 1^) are included in Suppl. Figures 7 and 8.

### Principle component analysis

**Fig. 2:**
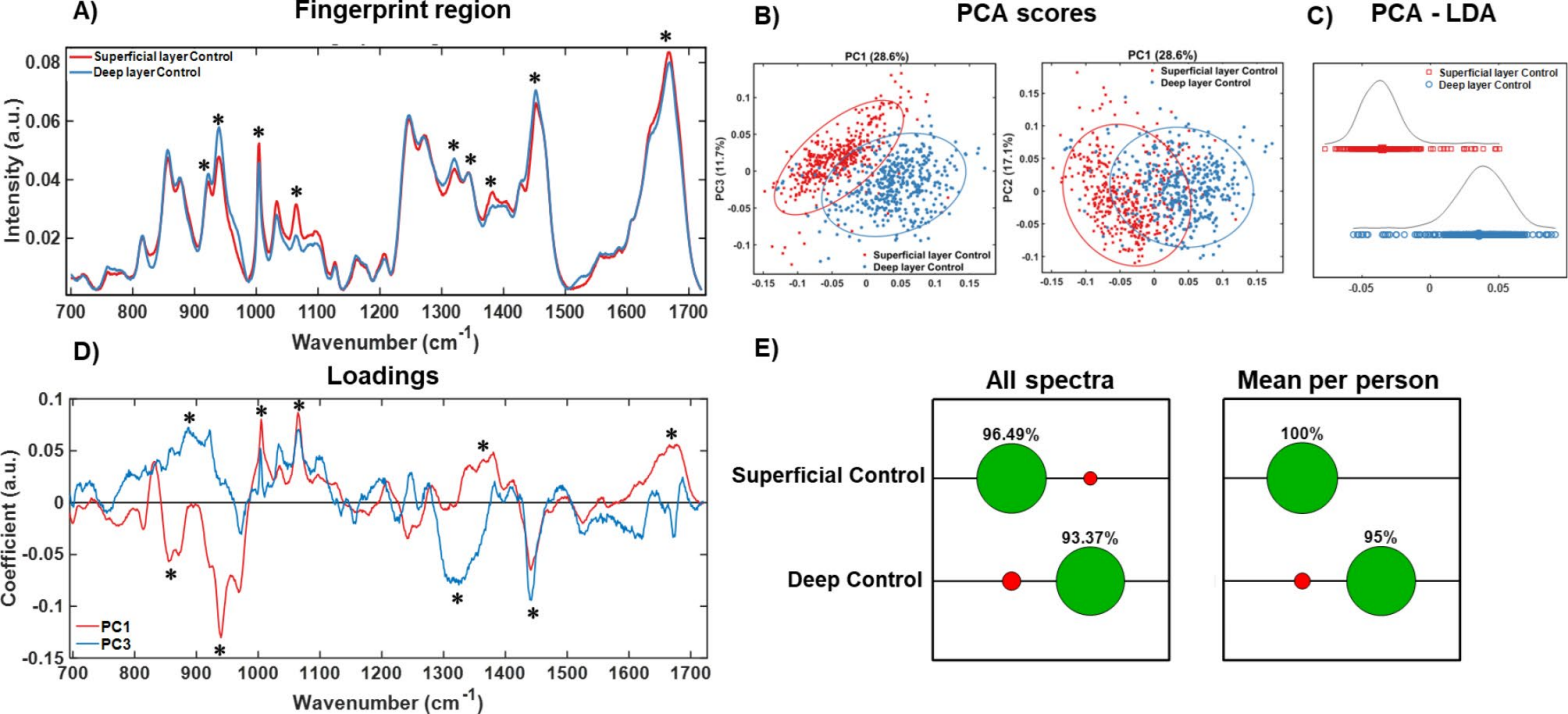
Raman spectroscopic analysis of superficial and deep layers of healthy cartilage. (**A**) Fingerprint region (700 to 1720 cm^− 1^) of RS of superficial (red) and deep (blue) layers of healthy “control” cartilage. (**B**) 2-D PCA scatter plots show distribution of superficial and deep layer spectra along PC1, PC2 and PC3 axes. (**C**) LDA analysis shows further separation of superficial (red) and deep (blue) layers based on class labels. (**D**) PC1 and PC3 loadings from the PCA. (E) Confusion matrices show the classification accuracy with correct classifications indicated in green and mis-classifications indicated in red of spectra from superficial or deep layer cartilage using all individual spectra and average spectra per patient. *N* = 19 (male *n* = 9, female *n* = 10). Asterisks (“*”) in (**A**) refer to “*” in (**D**) to highlight the main spectral peaks that contribute to PCA in (**B**).

**Fig. 3:**
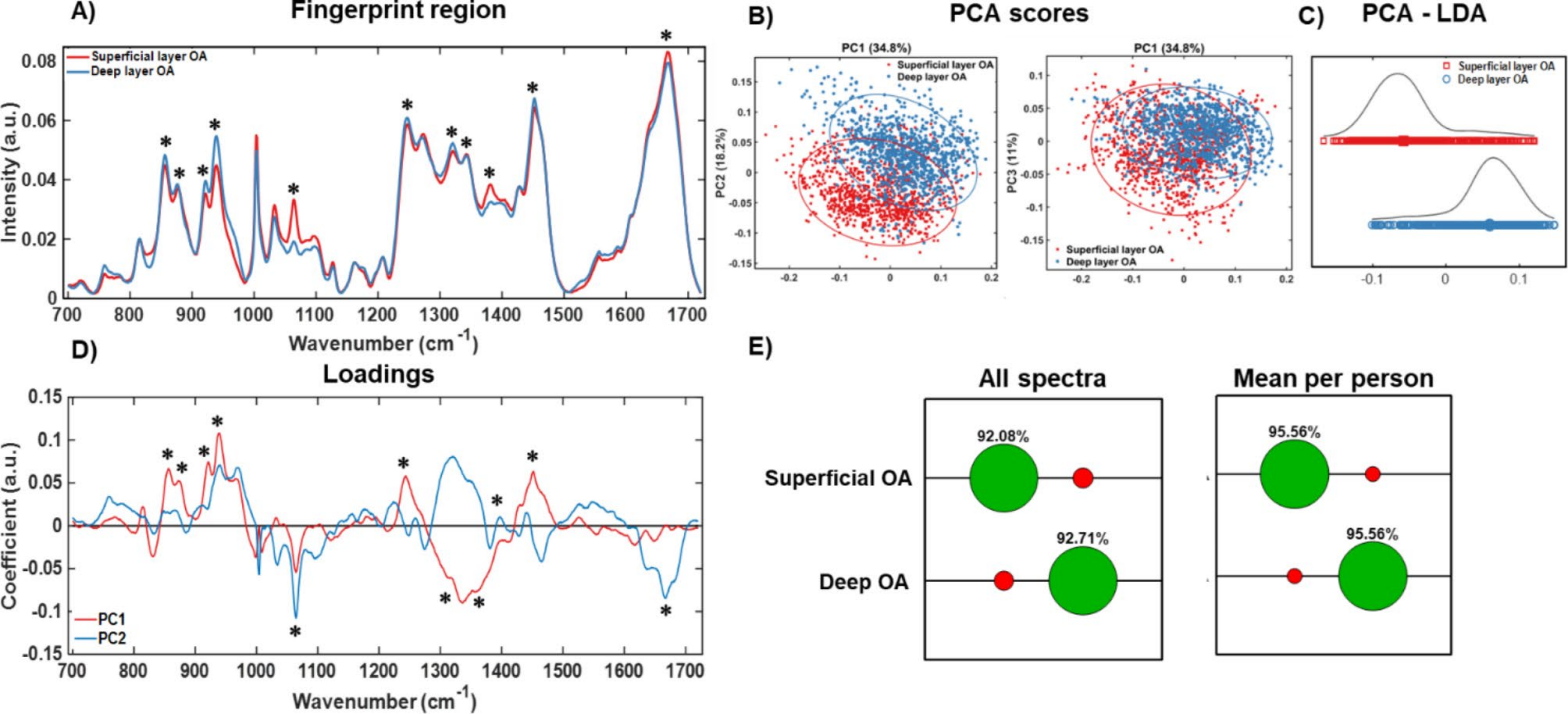
Raman spectroscopic analysis of superficial and deep layers of osteoarthritic (OA) cartilage. (**A**) Fingerprint region (700 to 1720 cm^− 1^) of RS of superficial (red) and deep (blue) layers of OA cartilage. (**B**) 2-D PCA scatter plots showing the distribution of superficial and deep layer spectra along PC1, PC2 and PC3 axes. (**C**) LDA analysis showed clear separation of superficial (red) and deep (blue) layer spectra into the respective classes. (**D**) PC1 and PC2 loadings corresponding to PCA scatter plot shown in (**B**). (**E**) Confusion matrices show correct classifications (green) and misclassifications (red) of superficial and deep layer spectra from OA cartilage samples using all individual spectra and average spectra per patient. *N* = 45 (male *n* = 21, female *n* = 24). Asterisks (“*”) in (**A**) refer to “*” in (**D**) and point to spectral peaks contributing to PCA scores in (**B**).

**Fig. 4:**
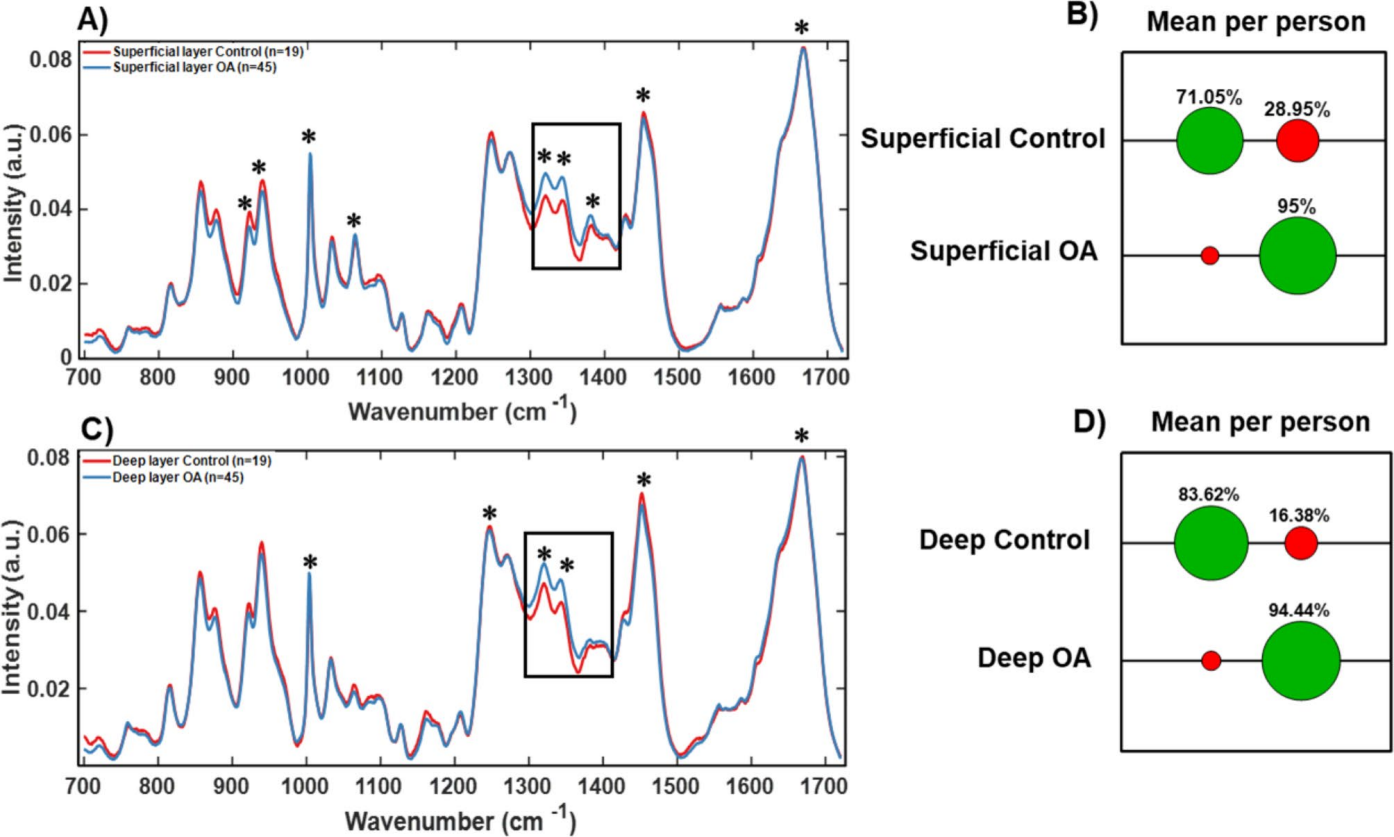
Raman fingerprint for OA diagnosis of articular cartilage. (**A**) RS of superficial layer and (**C**) of deep layer from Control (red) vs. OA (blue) cartilage. Boxed regions show the region with the highest difference for OA and contains 1320/1345 cm^− 1^ collagen modes and 1380 cm^− 1^ sGAG peak identified by asterisks. Confusion matrices in (**B**) and (**D**) show classification accuracy with correct assignments indicated in green and mis-assignments indicated in red for average spectra per patient. Additional “*” in (**A**) and in (**C**) refer to loadings in Suppl. Fig.S2 and indicate spectral peaks that contribute to PCA scores. Suppl. Fig. S2 also includes PCA-LDA, PC scores and Confusion matrices for all spectra.

**Fig. 5:**
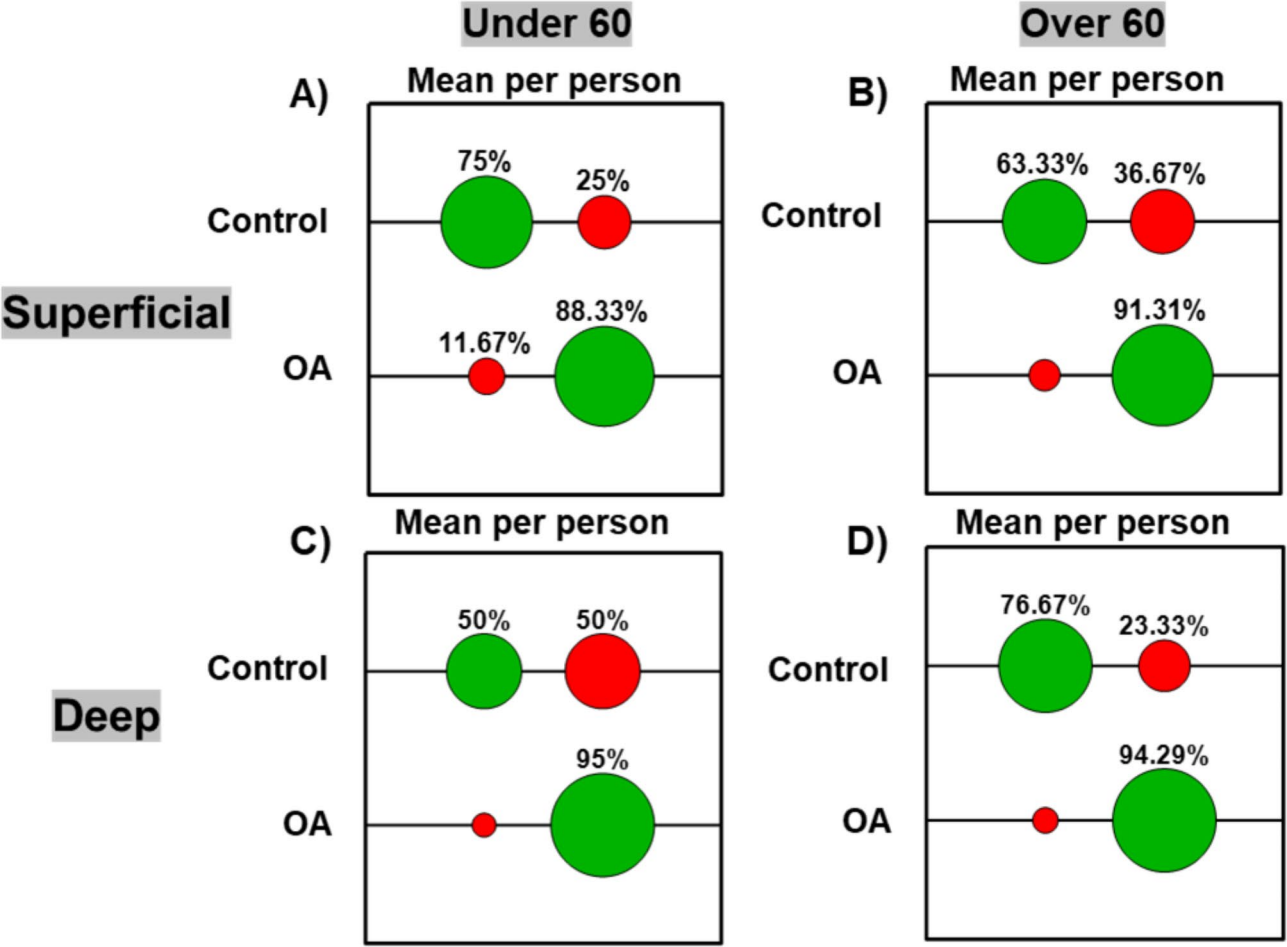
Effect of age on Raman spectral signatures of superficial and deep cartilage layers. Confusion matrices based on Raman spectra for OA diagnosis in under − 60 and over − 60 patient age groups (mean spectra per person). Superficial layer (**A**) and Deep layer (**C**) in under 60 cohort (control *n* = 5, OA *n* = 13). Superficial layer (**B**) and Deep layer (**D**) in over − 60 cohort (control *n* = 14, OA *n* = 32). Confusion matrices demonstrate percentage classification accuracy (green) and mis-classifications (red) based on SVM analysis of RS. RS, PCA, PCA-LDA, PCA scores and SVM on all spectra analysis are included in Suppl. Fig. S5 and S6.

Principal Component Analysis (PCA) was performed using the IrootLab plugin (0.15.07.09-v) for MATLAB R2021b^47^, all individual spectra and average per patient spectra were included into the analysis for characterising the heterogeneity of both superficial and deep layers of femoral heads (Figs. 2 and 3). OA diagnosis included analysis based on average spectra per patient with SVM in main figures (Figs. 4 and 5), with PCA, PCA-LDA and SVM for all spectra in Suppl. Fig. S2, S5 and S6. All spectra were carefully background subtracted using a fifth-order polynomial, and the ends of each spectra were anchored to the axis using the rubber band function. Spectra were smoothed by wavelet denoising before vector normalization (an example of data processing is shown in Suppl. Fig. S1). Data was trained-mean-centred for PCA to provide a valid decomposition of the original data. PCA was then carried out with 10 PCs. Percentages that give the portion of the data accounted for in each PC (eigenvector) are displayed in Suppl. Fig. S2, S5 and S6. The PC loadings relate to the variation in the spectra to PC scores and thus indicate which regions in the Raman spectra make the highest contribution to PCA. In addition to the first principal component (PC1) that has the highest score and represents the maximum variance in the data, the PC with the next highest score was used to model the systematic variation of the data set^48^. Thus, if PC3 score was higher than PC2 then that was used. The scores for each of the 10 PCs are shown in Suppl. Fig. S2, S5 and S6.

### Linear discriminant analysis

Linear Discriminant Analysis (LDA) was used to model differences within the RS data from superficial and deep layers of the cartilage samples following PCA analysis. PCA is an unsupervised method that maximizes the variation across the whole data set without considering the classes, if any, within a dataset. PCA-LDA cascade considers assigned classes and tries to maximize variation between them to give clustering and was used when PCA analysis alone failed to separate the data classes. While an unsupervised method is ideal for diagnosis, a supervised method such as PCA-LDA is likely to be closer to clinical application where a preliminary classification accuracy through clinical assessment or symptoms is likely to exist. Supplementary figures contain LDAs not included in the main figures as indicated in the Results sections.

### Support vector machine (SVM)

A supervised machine-learning classification was carried out via application of a Support Vector Machine (SVM) to assess the predictive capability of the Raman spectra when assigning cartilage to a particular group. This was carried out following a protocol within the Irootlab toolbox, established by its creator (SVM (studylib.net)^47^. Confusion matrices described the accuracy of a binary classification for models such as “diseased” vs “control”, “superficial” vs “deep” etc.” Further spectral pre-processing was carried out under the following protocols: “2nd-order SG Differentiation, cascaded to Vector Normalization”, then “Normalization to “0–1” range”. To account for the differences in the number of OA and Control sample numbers, Gaussian fit for unbalanced classes was applied, then the SVM classifier was built with a 5-fold k-cross validation with “Random seed”, and its optimal parameters for “c” and “gamma” ascertained through an automated grid search. Under these optimal parameters, a “Rater” could then be applied to the dataset, split for training and testing, with results described through confusion matrices to show prediction accuracy of the SVM model.

The all spectra (≥ 20 per patient per side of cartilage) from each patient was included into the analysis to include features in the spectra that will inform on the heterogeneity of OA across different parts of femoral heads. Average spectra per patient was used to inform on disease diagnosis. Both analyses are included in the relevant figures and in the Supplementary section.

### Multimodal imaging

Representative cartilage samples that were also analysed by Raman were imaged with multimodal imaging to complement the findings of Raman spectroscopic analysis and to explore correlations to changes in architecture and arrangement of lipid structures (including cells) and collagen. The same samples were used but not exactly the same areas as this were to explore whether there were any obvious observable changes. A detailed imaging study of cartilage samples and analysis is underway and will be reported in the near future. In this work to get preliminary correlations both transverse and longitudinal sections were taken. Transverse sections (from top of the cartilage to the bone) were taken with a scalpel blade and included superficial, middle and deep cartilage layers. Cartilage slices were washed with dH_2_O and positioned between two round cover slips (0.9 mm diameter, 0.17 thickness, Fisher) in a drop of dH_2_O fixed in a round ring sample holder (Attofluor™ Cell Chamber for microscopy Catalog number: A7816). Alternatively, cartilage slices were trimmed longitudinally with a razor blade to create flat surfaces and imaged at the top (superficial) and bottom (deep) surface; Raman spectra were also taken from the top and the bottom. The bottom layer samples were inverted on the sample stage for imaging and for Raman spectroscopy to acquire signals from the bottom side (closest to bone) of the bottom layer.

Images of cartilage slices were acquired using a home-built system which enabled image acquisition with coherent anti-Stokes Raman scattering (CARS), SHG and TPF, simultaneously as described by our lab before^49^. This multimodal laser scanning microscope employed ScanImage 5.1 (Vidrio Technologies) for image acquisition^50^. Briefly, for CARS imaging the fundamental of a fibre laser (1031 nm, 2 picosecond, 80 MHz, Emerald Engine, APE) was used as a Stokes beam, and the output of an optical parametric oscillator (OPO) (APE, Levante Emerald, 650–950 nm) which was synchronously pumped by the second harmonic (516 nm) of an AeroPulse fibre laser (NKT Photonics), was used as a pump beam. The multimodal platform utilises 2 ps pulses to provide multimodal capability with CARS, SHG and TPF simultaneously. Non-linear interactions increase with shorter pulse-widths; however, with CARS since vibrational line-widths are of the order of 10 cm^-1^, 2 ps pulse-widths are near ideal to ensure efficient excitation and reduction of non-resonant background and at the same time limits any compromise of signal generation with other non-linear modalities such as SHG and TPF. The two beams were made collinear and then coupled through a galvanometric scanner to an inverted microscope (Nikon Ti-E) configured for epi-detection. Their temporal overlap was controlled with a delay line. For imaging lipids in the cartilage slices, the C–H stretching mode at 2845 cm^-1^ was targeted, and for this the OPO was tuned to 797.5 nm. The total incident power on the sample was approximately 60 mW. The SHG (400 nm) and TPF signal (550 nm) was collected with the same OPO beam at 797.5 nm simultaneously with CARS in a 3-channel detection setup equipped with photomultipliers (PMTs). Each sample was imaged using a x40 (1.15 NA) water immersion objective, with 3 or 6 optical zoom using galvanometric scanning, and acquisition time of 16 ms per line for a 512 × 512 pixel image. The detection setup included dichroic beamsplitters with cut-offs at 442 nm (Semrock Di02-R442) and 594 nm (Semrock Di02-R594)^49^.

## Results

### Methodology and workflow

Raman Spectroscopy (RS) was carried out to identify signatures of the superficial and deep layers of human cartilage from femoral heads^3^. Cartilage from the femoral head of osteoporotic (OP) donor patients was used as a “healthy” control. Control femoral heads from OP patients displayed a smooth uninterrupted cartilage surface and a fractured bone area at the “neck” of the sample. Osteoarthritic (OA) femoral heads were predominantly Grade III-IV, with the cartilage completely absent from the weight bearing regions in most of the samples analysed. A few OA samples represented earlier stages of OA but were clearly identified as OA by the clinical team. In order to investigate chemical changes prior to complete disappearance of cartilage only intact/thick cartilage was removed and used for Raman spectroscopy, wherever possible. All analysed samples were age and gender matched where possible (Table 2). The mean age for all the groups was around 70 years old. Mean age for the control samples was around 2 years higher than for OA samples (71.3 and 72 years old for female and male controls, while it was 68.9 years for female OA and 69.6 years old for male OA).

The workflow involved the acquisition of Raman Spectra and multimodal imaging from the superficial and deep layers, followed by analysis by principal component and linear component analysis and classification accuracy (Fig. 1). Results for superficial and deep layer healthy cartilage (controls) samples are shown to illustrate the workflow. Raman spectra and corresponding PCA loadings between 700 cm^−1^ to 1720 cm^-1^ are shown. PCA (unsupervised) and scatter plots using PC1 and PC3 scores are shown together with PCA cascaded into LDA (supervised). PCA-LDA scatter plot shows clear separation of the classes into “Superficial” and “Deep” layers along the LD1 axes (Fig. 1(2)). Confusion matrices were created from SVM analysis where the size of the circles indicates classification accuracy (green) and misclassification accuracy (red), respectively. Cross-sections of representative cartilage samples were imaged using CARS/SHG/TPF multimodal imaging to observe if any chemical and structural changes can correlate and corroborate Raman spectral analysis. CARS was used to image structures rich in lipids by using the symmetric stretch vibration of –CH_2_ bonds at 2845 cm^-1^; SHG was used to image collagen fibres and TPF was used to image autofluorescence emitted at 550 nm that highlights cells rich in FAD/NADH^51,52^. Representative images are shown in Fig. 1(3) wherein collagen fibres can be seen around a cluster of 4 cells with different levels of lipids and autofluorophores.

### Characterisation of Raman signatures in healthy cartilage

The methodology described above was applied to superficial and deep cartilage layers of each slice in healthy cartilage samples (Fig. 2). All spectra taken from the superficial and deep layers were compared to identify differences between their “Raman fingerprint”. The mean spectra per class (Superficial and Deep layer) are shown in Fig. 2 (A) to clearly show differences in the spectral regions. The mean spectra with standard deviation for all individual spectra, average spectra per patient, all individual spectral recordings, and spectral data processing steps, are shown in Supplementary Fig. S1 to demonstrate quality of the recordings and contribution of background fluorescence from the biological tissue samples. A prominent difference between the superficial and deep layers at a sulphated glycosaminoglycan (sGAG) peaks of 1064 cm^-1^ and 1380 cm^-1^ was observed which are indicative of different mechanical and biochemical matrix properties as shown in Klein et al. and others^4,38,53,54^. The concentration of aggrecan, which is a large proteoglycan and contains large chondroitin sulphate chains (1064 cm^-1^ peak) is much higher in the superficial layer to accommodate extracellular water retention capacity to counteract external pressures of the loads onto the cartilage^55^. Seven other peaks were also identified (asterisks Fig. 2 (A) and in Loadings in Fig. 2 (D)), corresponding to 877 cm^-1^ (Hydroxyproline), 921 cm^-1^ (Proline), 938 cm^-1^ (Hydroxyproline), 1004 cm^-1^ (Phenylalanine), 1320 cm^-1^ (Collagen wagging)/1345 cm^-1^ (Collagen twisting), 1451 cm^-1^ (Collagen type II/Amide III), 1666 cm^-1^ (Collagen/Amide I). These Raman bands were the main contributors to PC1, PC2 and PC3 scores in Fig. 2 (B) as evident from Loadings in Fig. 2 (D), except for the 1004 cm^-1^ Phenylalanine peak which displayed variability within spectral readings. LDA analysis clearly delineated superficial and deep layer spectra on the LD1 axis with negligible overlap in Fig. 2 (C). Loadings are shown for PC1 and PC3 in Fig. 2 (D); the latter had a higher score than PC2 and it can be seen in Fig. 2 (B) that separation is better along the PC3 axis compared to PC2. As shown in the confusion matrix based on SVM an accuracy of 97% and 93% was achieved for superficial and deep layers using all spectra and, 100% and 95% on using mean spectra per patient, respectively, in Fig. 2 (E). The scores from Confusion matrices suggest that Raman can very accurately identify both Superficial and Deep layers in “healthy” cartilage with possibly very little heterogeneity present within and between cartilage samples used here.

Additionally, Raman spectra from the C-H stretching region (2500–3300 cm^-1^) also gave very high classification accuracy of 95% and 90% for Superficial and Deep layers of healthy cartilage, respectively (Suppl. Figure 7).

### Characterisation of Raman signatures in OA cartilage

OA cartilage samples were analysed similar to “healthy” samples to find out if there were any differences in Raman signatures between the different layers. The spectra and analysis results in Fig. 3 have shown prominent differences between superficial and deep layers at the sulphated glycosaminoglycan (sGAG) peaks of 1064 cm^-1^ and 1380 cm^-1^ and hydroxyproline peak of 938 cm^-1^, with the latter peak potentially representing a change in collagen structure in OA. Six other peaks (indicated with asterisks in Fig. 3 (A) and (D) were found to make significant contributions to PC1, PC2 and PC3 loadings; the peak positions and likely assignments were as follows: 855 cm^-1^ (Proline/Collagen backbone), 877 cm^-1^ (Hydroxyproline), 921 cm^-1^ (Proline), 938 cm^-1^ (Hydroxyproline), 1320 cm^-1^ (Collagen wagging)/1345 cm^-1^ (Collagen twisting), 1451 cm^-1^ (Collagen type II/Amide III), 1666 cm^-1^ (Collagen/Amide I).

LDA analysis clearly separated the superficial and deep layer spectra along the LD1 axes (Fig. 3 (C), as observed for healthy cartilage samples (Fig. 2). The mean spectra with standard deviation for all individual spectra, average spectra per patient and all individual spectral recordings are shown in Suppl. Fig. S1. SVM analysis of the spectra gave 92% and 93% accuracy for superficial and deep layers using all spectra and 96% for both layers on using mean spectra per patient as shown in the confusion matrix (Fig. 3 (E)). Similar to “healthy” controls, RS can very accurately identify both Superficial and Deep layers in OA cartilage with possibly very little heterogeneity present within and between cartilage samples used here, also, hinting that OA does not alter chemical composition of cartilage to the point where “Fingerprint” of the layers becomes homogeneous throughout.

“Raman spectra from the C-H stretching region (2500–3300 cm^-1^) gave, although slightly lower than in “Healthy” control cartilage, very high classification accuracy of 90% and 88% for Superficial and Deep layers of OA cartilage, respectively (Suppl. Figure 7).

### Diagnostic signatures of OA based on Raman spectroscopy of superficial and deep layer cartilage

To determine the diagnostic potential of Raman signatures at different depths, the spectra from OA and control superficial and deep layers of cartilage were compared and analysed (Fig. 4, Suppl. Fig. S2). Results indicated that the main peaks that differentiated superficial and deep cartilage layers were 1320 cm^-1^ (Collagen wagging) and 1345 cm^-1^ (Collagen twisting) peaks of collagen modes. This is consistent with remodelling of “fibrillar structures” of collagen in OA^34,39^ and characterised by initiation of synthesis of type IIa and III procollagens that are less efficient^16^. In addition, other 7 peaks were observed to be key in distinguishing OA from healthy cartilage in the Raman Spectra of the superficial layer, specifically 921 cm^-1^ (Proline), 938 cm^-1^ (Hydroxyproline), 1004 cm^-1^ (Phenylalanine), 1380 cm^-1^ and 1064 cm^-1^ (sulphated glycosaminoglycan (sGAG), 1451 cm^-1^ (Collagen type II/Amide III) and 1666 cm^-1^ (Collagen type II/Amide I). Proline, hydroxyproline, phenylalanine and sulphated glycosaminoglycans are known to undergo changes in OA^34,35,38,40,43^. Changes in sGAG are indicative of osmolarity/hydration changes that occur in cartilage with disease^6,34,55–57^, while amide III peaks relate to amounts of total collagen in the samples. The spectral signatures from superficial and deep layers, respectively, of OA with corresponding healthy controls were compared. Although 1004 cm^-1^ phenylalanine and the 1245 cm^-1^ collagen peaks were altered, in contrast to the work of Gaifulina et al.^58^ we observed that the differences were not as significant. In addition, it was observed that the sulphated glycosaminoglycan (sGAG) 1064 cm^-1^ peak was significantly different between control and OA samples in Superficial layer (Suppl. Fig. S2, Table S3). However, in the studies of Gaifulina et al.^58^, the differences between different layers of cartilage were not considered nor depth correlated comparisons carried out between control and OA samples and may account for the differences observed in the current study.

It is clear from the spectral loadings that sGAG (1380 cm^− 1^ peak) was increased in OA superficial layer compared to controls (Suppl. Fig. S2 (1(i), Table S3). The 1380 cm ^− 1^ peak has been assigned to sGAGs^43^ indicating an unstable cartilage matrix in OA. Additionally,1245 cm^− 1^ peak Loadings were altered in both superficial and deep layers consistent with the remodelling/change of direction of collagen fibres^35,39,59^ (Fig. 4 (C), Loadings in Suppl. Fig. S2 (1(i) and 2(i)). PCA, LDA and Loadings analysis is included in Suppl. Fig. S2.

Further analysis by SVM allows classification accuracy of healthy cartilage of 71% and 67% on using all spectra (Suppl. Figure 2 (1(v) and 2(v)) and 71% and 84% on using mean spectra per patient for superficial and deep layers, respectively (Fig. 4 (B) and (D)). OA cartilage was identified from the dataset with higher accuracy than control of 88% and 95% on using all spectra (Suppl. Figure 2 (1(v) and 2(v)) and 95% and 94% on using mean spectra per patient analysis for superficial and deep layers, respectively (Fig. 4 (B) and (D)). We found that SVM analysis gave similar or higher classification accuracy when using average spectra per patient compared to when using all spectra for superficial control layer. This suggests that although average spectra per patient is accurate in diagnosis of OA, it possibly misses or does not include the heterogeneity of the disease between different tissue areas. Mapping of the separate areas of cartilage on femoral heads is currently being explored in the lab and will address this question.

Additionally, Raman spectra from the C-H stretching region (2500–3300 cm^-1^) also gave very high classification accuracy of OA cartilage in both Superficial and Deep layers of 88% and 84% respectively (Suppl. Figure 8 (1(v), 2(v)). Although, these results should be taken with caution as the control samples had low accuracy of prediction of 49% and 62% for Superficial and Deep layers respectively (Suppl. Figure 8 (1(v), 2(v)).

### Raman spectroscopy identifies chemical fingerprint of age-related changes in cartilage from controls and OA

Age is an important factor in the development of OA. Hence, we also examined whether there were any age-related differences in the different layers in healthy and OA cartilage. Samples were divided into two age groups (under − 60 and over − 60). The groups were selected to provide a cohort aged below 60 years of age (“young”) and an “older” cohort, over 60 years of age given the prevalence of OA increases in older patients (Fig. 5).

The under − 60 patient cohort, included 5 healthy and 13 OA samples and the over 60 patient cohort comprised of 14 healthy and 32 OA samples. Under − 60 OA samples were correctly classified with high accuracy of 88% and 95% on using average spectra per patient (Fig. 4 (A) and (C)) and with 85% and 96% accuracy on using all the spectra for superficial and deep layers, respectively (Suppl. Fig. S5 (1(v)) and S6 (1(v)). Similarly, over − 60 OA patient samples were correctly classified with 91% and 94% on using average spectra per patient (Fig. 4 (B) and (D)) and with 92% and 91% on using all the spectra for superficial and deep layers, respectively (Suppl. Fig. S5 (2(v)) and S6 (2(v)) suggesting that Raman is very sensitive in identifying OA cartilage in both “young” and “older” patients. However, the classification accuracy of “healthy” control cartilage was less accurate with 75% for under − 60 and 63% for over − 60 from spectra taken from superficial layer, using average spectra per patient analysis. “Healthy” deep layer cartilage was classified correctly with only 50% for under − 60 for both all and average spectra per patient, while spectra from over − 60 healthy samples were correctly classified with higher accuracy of 67% and 77% using all spectra and average spectra per patient, respectively (Fig. 5, Suppl. Fig. S5 and S6). RS, PCA, PCA-LDA, PCA scores and SVM on all spectra analysis are included in Suppl. Fig. S5 and S6. The classification accuracy was low for spectra from under − 60 healthy samples potentially due to lower sample numbers (*N* = 5). This analysis shows that despite lower accuracy in identifying “healthy” samples, accuracy of diagnosis of OA cartilage was ≥ 90% for both “young” and “older” patients.

### Effect of gender on Raman fingerprint of different cartilage layers

**Fig. 6:**
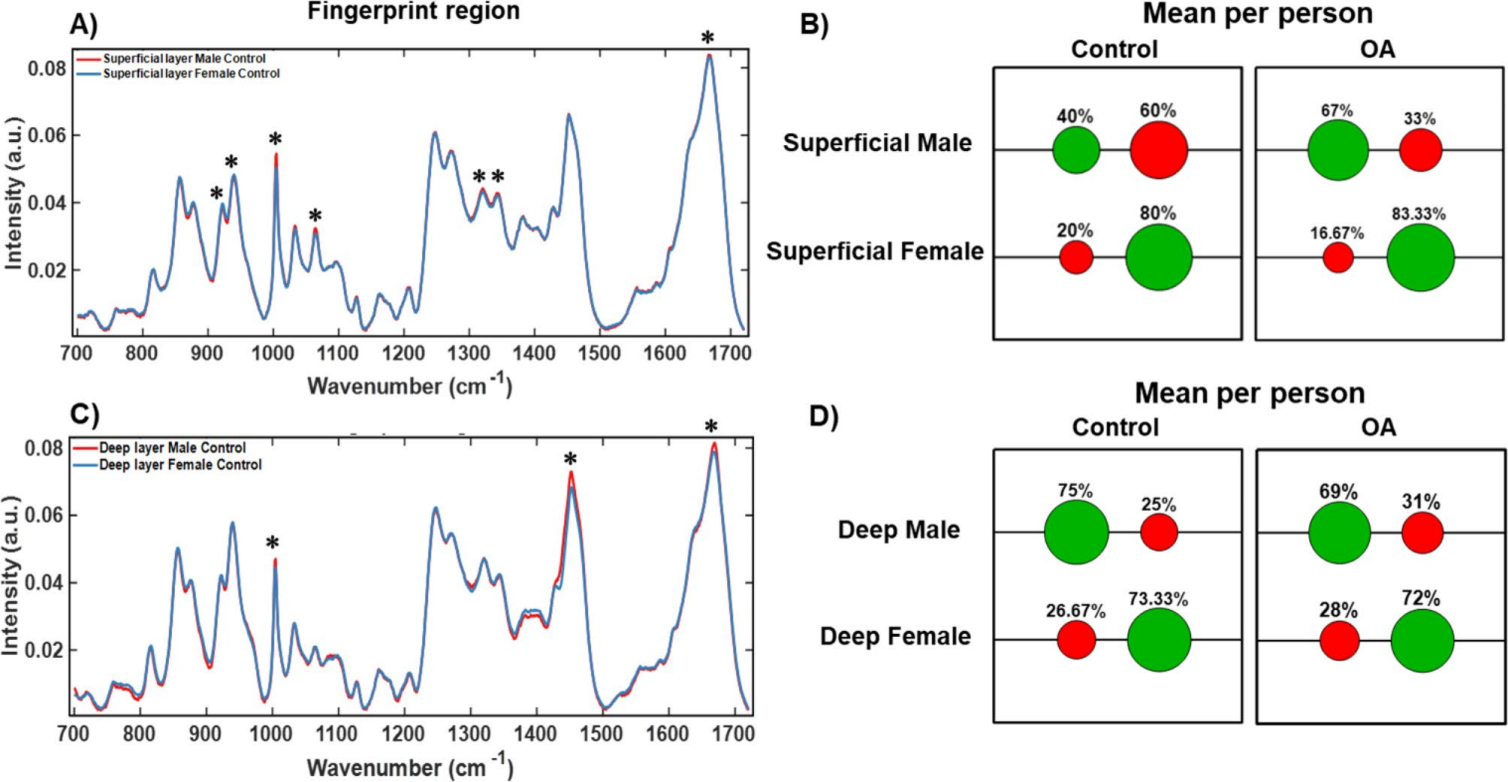
Effect of gender on Raman signatures in different layers of healthy articular cartilage. (**A**) RS of superficial layer and (**C**) of deep layers from healthy cartilage samples of male (red) vs. female (blue). Confusion matrices in (**B**) and (**D**) demonstrate positive (green circles) and negative (red circles) scores in assignments to Male or Female samples in controls and OA cartilage. Asterisks (“*”) in (A) refer to Loadings in Controls in Suppl. Fig. S3 and show spectral peaks that contribute to PCA scores. Suppl. Fig. S3 also includes PCA, PCA-LDA, PC scores and Confusion matrices for all spectra for Control samples. Suppl. Fig. S4 includes RS, PCA, PCA-LDA, PC scores and Confusion matrices for all spectra for OA samples.

Following establishment of the underlying chemical basis of OA diagnosis based on superficial and deep layers of cartilage tissue an analysis for gender dependence in the different layers of cartilage was undertaken. Raman spectra from gender matched healthy cartilage samples were compared. The mean Raman spectra from the superficial and deep layers in healthy cartilage are shown in Fig. 6 (A) and (C). The main peaks 921 cm^-1^ (Proline), 938 cm^-1^ (Hydroxyproline), 1004 cm^-1^ (Phenylalanine), 1064 cm^-1^ (sulphated glycosaminoglycan (sGAG), 1320 cm^-1^ (Collagen wagging) and 1345 cm^-1^ (Collagen twisting) modes, 1666 cm^-1^ (Collagen/Amide I) that contributed to PCA loadings (Suppl. Fig. S3). Importantly, these peaks also distinguish control and OA samples as shown in Fig. 4, except for the 1666 cm^-1^ peak that is indicative of total protein content in cartilage. The Raman spectroscopy data, the PCA and LDA analysis (Suppl. Fig. S3), demonstrated fewer differences in chemical composition between male and female cartilage samples. The peaks corresponded to protein composition and structure of collagen fibres, which may be more sensitive to changes occurring in OA. Interestingly, proline, sGAG and collagen structural modes contributed to PCA loadings for the superficial layer but not for the deep layer (Suppl. Fig. S3). The large overlap in PCA scores (Suppl. Fig. S3) in the male and female samples indicated a high level of similarity between the spectra. Despite this, the classification accuracy between male and female control cartilage samples was 80% and 73% for superficial and deep layer in female for mean spectra per patient (Fig. 6 (B) and (D). For males, the classification accuracy for mean per person data was 75% for the deep layer and only 40% for the superficial layer (Fig. 6 (B) and (D). As it is much more challenging to obtain samples with “healthy” cartilage, the last result might be improved with higher number (“N”) of samples in future work. Given the degree of overlap and reduced classification accuracy with gender for males, the current data suggest OA results in significant changes in chemical composition across both genders as evident from the analysis in the previous section (Fig. 4). We also compared classification accuracy of gender assigned OA samples. Similarly to controls, the classification accuracy for mean per person data for females was higher than for males (Fig. 6 (B) and (D)). Accuracy of predicting female OA cartilage was 83% and 72% for superficial and deep layers respectively on using mean spectra per patient. For males, the classification accuracy for mean per person data was 67% for the deep layer and 69% for the superficial layer. Raman spectra for gender assigned OA cartilage, PCA, PCA – LDA analysis and corresponding Loadings for both control (Suppl. Fig. S3) and OA samples (Suppl. Fig. S4) are included in Supplementary section.

### Multimodal imaging of cross sections of cartilage from superficial to deep layer

**Fig. 7:**
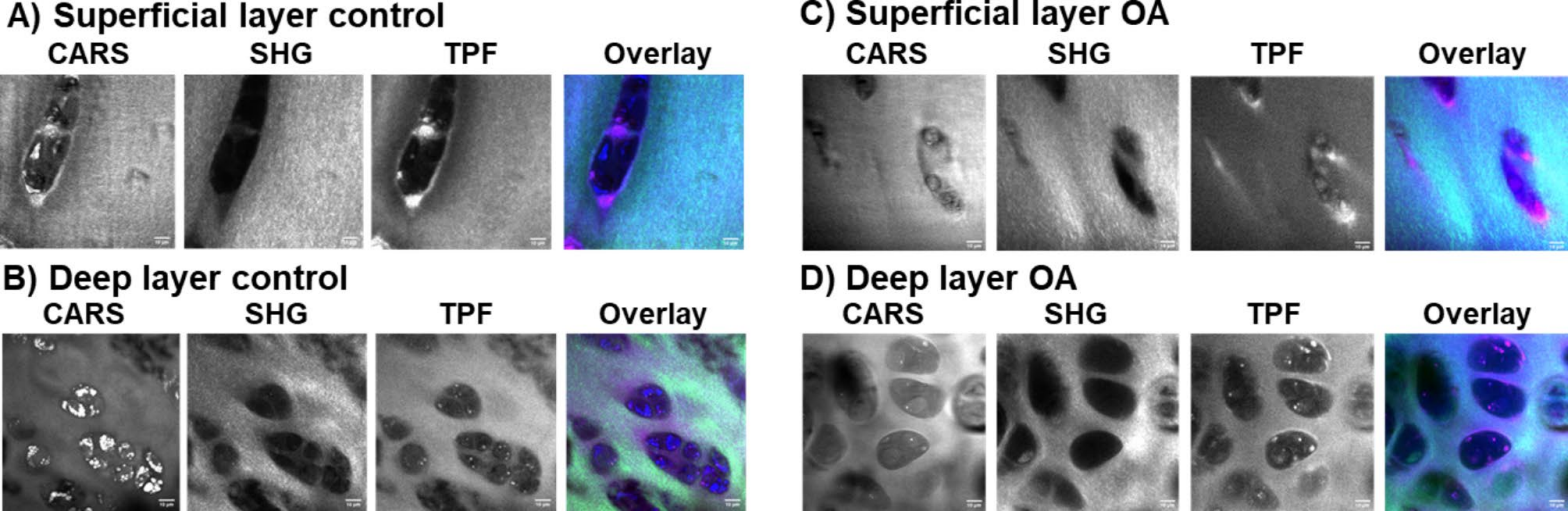
Multimodal imaging of the longitudinal direction (from the top and the bottom) sections of cartilage. (**A**) Superficial and (**B**) Deep layer from a control patient sample (M84 Control); (**C**) Superficial and (**D**) Deep layer from an OA patient sample (F44 OA). CARS at 2845 cm^− 1^ showing lipids, cell membranes and matrix, SHG (400 nm) showing type II collagen fibres in cartilage, TPH (550 nm) showing autofluorescence of collagen and other biological molecules, overlay with CARS (red), SHG (green) and TPF (blue). The scale bar is 10 μm.

**Fig. 8:**
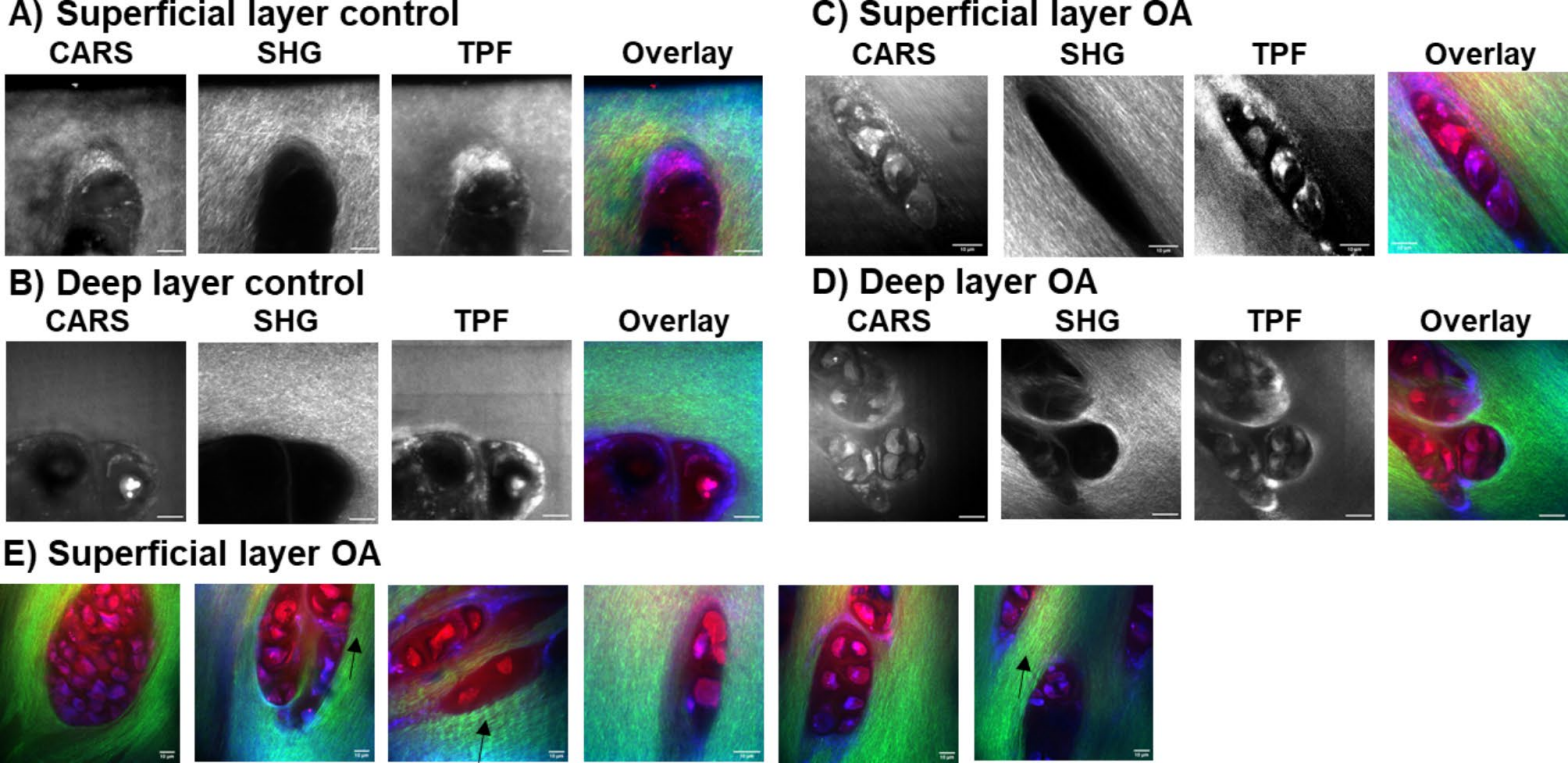
Multimodal imaging in the transverse (perpendicular to the bone) samples of cartilage. (**A**) Superficial (M88 Control) and (**B**) Deep (F58 Control) layer from a control sample; (**C**) Superficial (F69 OA) and (**D**) Deep (M62 OA) layer from an OA sample. CARS at 2845 cm^− 1^ showing lipids, cell membranes and cartilage matrix, SHG (400 nm) showing type II collagen fibres, TPH (550 nm) showing autofluorescence of collagen and other biological molecules, overlay with CARS (red) of cells, SHG (green) of collagen and TPF (blue) of autofluorescent biological molecules. (**E**) Representative composite images of cells clustering in OA in Superficial layer and “wavy” structure of collagen fibers shown by arrows, overlay as in above. The scale bar is 10 μm.

Following Raman spectral analysis, we examined representative samples using multimodal imaging for any correlation in spectra with a focus on collagen and peaks generated by lipid and cartilage matrix proteins. CARS (2845 cm^− 1^), SHG (400 nm) and Two photon fluorescence (550 nm) images were taken in the transverse (vertical section perpendicular to the cartilage/bone interface) and in longitudinal direction from both the top (superficial layer) and bottom (deep layer) of the samples (as shown in Fig. 1) in 3 controls and 4 OA patients (Figs. 7 and 8) for a preliminary analysis. Cartilage slices were prepared with a razor blade and imaged in both directions to allow better visualization of the cells in the longitudinal direction (Fig. 7, CARS and TPF) and to achieve stronger clearer SHG signal from cartilage fibres in the transverse direction (Fig. 8, SHG).

In the longitudinal sections (Fig. 7), the collagen fibres in the SHG images appeared as discrete white streaks (bundles of tightly packed fibres) within a grey/white background and notably more abundant in superficial compared to deep cartilage layers (Fig. 7, SHG images). Outlines of the chondrocytes (cells) were distinguishable in CARS (lipid membranes of cells) and TPF (autofluorescence from pericellular components such as collagen VI and/or NADH/FADH) images. Lipid droplets inside the cells were present in both controls and OA samples, clearly seen in the deep layer (Fig. 7, CARS and TPF images in (B) and (D)). In Fig. 7 CARS image in (B) bright lipid droplets within chondrocytes in the deep layer of M84 control cartilage were not observed in any other samples and potentially represent a patient specific result unrelated to OA or control samples. We noted the shape and cluster appearance of the chondrocytes in the superficial (large, vertical to the surface and elongated) and deep (small, clustered, horizontal) layers differed in direction and size and were in agreement with published research on stained cartilage^16^. Similarly, representative images of the top (superficial layer) and bottom (deep layer) of cartilage in healthy and OA cartilage samples are shown in Fig. 8, taken from transverse thin sections of cartilage. CARS and TPF images were comparable to those shown in Fig. 7, however, SHG images contained well defined collagen fibres in transverse compared to longitudinal direction cartilage slices, due to the alignment of the collagen fibres facing the laser longitudinally (Fig. 8) rather than perpendicularly (Fig. 7) as in the longitudinal sections. The superficial layer likely contains fibres that form a meshwork, with majority of fibres longitudinal to the surface of the joint, and hence, can be seen in both the longitudinal (Fig. 7, SHG images in (A) and (C)) and transverse directions with higher brightness in the later (Fig. 8, SHG images in (A) and (C)). The collagen fibres are aligned along the perpendicular direction to the bone and thus to the laser in the deep layer of cartilage in longitudinal sections and, hence, appeared diffuse (Fig. 7, SHG images in (B) and (D)). We looked at the features of the cartilage and, in particular, appearance of collagen fibres and chondrocytes. In the superficial layer images, the collagen fibres displayed a more wave-like phenotype in OA than in control samples as shown with the black arrows in Fig. 8 (E). CARS/SHG/TPF composite images in Fig. 8 (E) are shown as examples from different OA patients to illustrate observed masses of chondrocytes attributed to cell clustering (Fig. 8 (E), CARS and TPF, also in Deep layer OA in CARS and TPF in Fig. 8 (D) and “wavy” collagen fibres (SHG) (Fig. 8 (E) arrows in SHG), a signature of OA^16^. The “wavy” appearance of collagen fibres may offer a new diagnostic feature for OA and probably refers to the changes in collagen modes observed in Raman (1245 cm^− 1^ and 1272 cm^− 1^, 1320 cm^− 1^ and 1345 cm^− 1^, 1451 cm^− 1^). We attempted to extract further information from the brightness of the cartilage matrix (CARS, TPF) and parameters of the collagen fibres like their thickness, length and straightness but were unable to obtain reliable measurements that were consistent between the samples and within the samples from the same patient due to being unable to slice unprocessed cartilage to the same thickness without it being ripped by the cryostat blade and very tight packing of the collagen fibres inside the tissue. Such imaging-based signatures will be explored further with additional control and OA samples to overcome technical difficulties and appropriate analysis procedures. Nevertheless, the current observations with the representative multimodal imaging data contain clear changes in collagen in both superficial and deep layers across control and OA samples and indicate possible correlation with the Raman spectroscopic analysis.

## Discussion

Raman Spectroscopy was used to characterise osteoarthritic and non-osteoarthritic patient samples of cartilage derived from femoral heads post hip arthroplasty in the cartilage superficial and deep layers. Discrete differences in the signatures that are likely due to changes in collagen and other matrix biological molecules such as sGAGs were identified. Specifically, the vibrational peaks/modes corresponding to 855 cm^− 1^ and 877 cm^− 1^ (proline, prominent in collagen), 921 cm^− 1^ and 938 cm^− 1^ (hydroxyproline), 1064 cm^− 1^ and 1380 cm^− 1^ (sGAG), and 1245, 1272, 1320, 1345, 1451, 1666 cm^− 1^ (collagen) demonstrated significant changes between the different cartilage layers.

In agreement with our findings, changes in proline and hydroxyproline were previously reported in work investigating collagen degradation in animal and synthetic models of cartilage matrix, and in studies exploring proline/hydroxyproline as potential biomarkers of the OA, wound healing and other diseases of the connective tissues^57,60–62^.

In our studies, we detected alterations to 1245 cm^− 1^ /1272 cm^− 1^ and 1320 cm^− 1^ /1345 cm^− 1^ collagen bands in OA samples in both superficial and deep layers (except, 1272 cm^− 1^ changed only in the deep layer) suggesting remodelling and/or degradation of collagen throughout the cartilage. Interestingly, while 1320 cm^− 1^ /1345 cm^− 1^ bands were not identical between superficial and deep layers in both control and OA cartilage, 1245 cm^− 1^ band contributed to separation of layers in OA samples; neither 1245 cm^− 1^ /1272 cm^− 1^ differed between the layers in controls.

Changes in collagen structure were as expected and were previously reported in OA and other degenerative diseases in human knee cartilage (1241 cm^− 1^ /1269 cm^− 1^ amide III doublet)^39^, where ratio increased with the increased severity of the OA, likely, due to the increased degradation rates of collagen, in studies examining thermal and mechanical denaturation of collagen models^59^. Changes in collagen II modes at 1320 cm^− 1^, 1640 cm^− 1^ and 1670 cm^− 1^ were also detected in the above work, corresponding to 1320 cm^− 1^ and 1666 cm^− 1^ bands in our study, except, we did not observe the 1640 cm^− 1^ band^59^. Variable distribution of proline, hydroxyproline and collagen modes at 1244 cm^− 1^ /1270 cm^− 1^ amide III doublet in different layers of mature pig cartilage was also detected in the work by Bonifacio et al.^63^ confirming similar chemical structure of cartilage between mammalian species.

Differential composition of layers of cartilage matrix was observed from 1064 cm^− 1^ and 1380 cm^− 1^ peaks referring to components of the sGAG complex. In particular, 1064 cm^− 1^ chondroitin sulphate peak contributed to separation of spectra from superficial and deep layers of both control and OA cartilage. This difference was very pronounced and can be explored for Raman spectroscopy with a fibreoptics probes for patients’ diagnosis and treatment. These two bands, in addition, to bands referring to collagen type II above, can be explored as biomarkers to minimally invasive approaches, e.g. arthroscopy, also explored by Kumar et al.^34^ and to validate models of tissue-engineered cartilage^14,35,40,44^. In agreement with our Raman spectroscopy based findings, differential cartilage matrix composition^38^ and changes in OA^40,43^ were reported in multiple studies in humans, model animals and tissue engineered cartilage^54^, e.g. in humeral cartilage of the shoulder joint^43^.

Our Raman spectroscopic measurements and multivariate analysis indicated clear differences between the chemical composition of superficial and deep layers of cartilage (Suppl. Tables S1, S2 and S3). Support vector machine analysis was performed on both mean spectra per patient and all collected spectra per patient for a group of samples to enable OA diagnostics and detect heterogeneity within and between individual patients, respectively. While different layers of cartilage were detected with high accuracy of 93-100% for both types of analysis, analysis using mean spectra per patient gave higher accuracy of detection both for different cartilage layers and separating OA from control cartilage, suggesting that this method captured the differences within cartilage of individuals. Comparison of superficial layer between control and OA samples indicated an 85% and 88% classification accuracy achieved for OA from all and mean spectra respectively. OA in the deep layer cartilage was predicted with 84% and 94% accuracy from all and mean spectra respectively. Accuracy of the identification of individual layers, OA, age and gender specific cartilage is summarised in Supplementary table S1.

High wavenumber region of RS of 2500 to 3300 cm^− 1^ was shown by Gaifulina et al.^58^. to separate control and early stage OA cartilage from knee joints. In our work, we observed a 88-95% classification accuracy of the superficial and deep layers in both control and OA samples, and 86-90% classification accuracy of OA cartilage. However, accuracy of distinguishing control cartilage from OA samples was only 50-62% suggesting that a combination of methods of analysis and low (fingerprint) wavenumber regions should be considered along with the high frequency region.

The spectral differences between the superficial and deep layers showed less significant differences and reduced accuracy with gender, although, cartilage from males was identified with close to 80% accuracy for both layers except for the analysis based on mean spectra per patient which only had 40% accuracy for superficial layer. Female derived cartilage was identified with a stable 65–73% accuracy for both all spectra and mean spectra per patient analysis. In contrast, OA cartilage was identified with high accuracy of 85–96% in under-60 group and 91–94% in over-60 group, including both methods of analysis.

Critically, age-based analysis indicated the importance of analysing different cartilage layers. Analysis of the superficial layer produced a higher classification accuracy for healthy samples in the cohort of patients under 60 years of age, while deep layer analysis resulted in higher classification accuracy in the cohort of patients over 60 years of age.

Multimodal imaging techniques confirmed the correlation of changes in the structure and organization of the chondrocytes and collagen in the superficial and deep layers with the results obtained following Raman spectral analysis. The collagen fibres appeared diffuse in the deep layer of cartilage in longitudinal sections due to them being aligned along the perpendicular direction to the bone and thus to the laser polarisation in SHG imaging. In contrast, collagen fibres in the transverse layers of cartilage appeared the brightest in SHG imaging due to them being parallel to the laser source. We identified features specific to OA cartilage where collagen fibres displayed a wave-like phenotype and possibly offer a new diagnostic feature for OA. This may relate to the changes in collagen modes observed in Raman (1245 cm^− 1^ and 1272 cm^− 1^, 1320 cm^− 1^ and 1345 cm^− 1^, 1451 cm^− 1^). Additionally, we observed masses of chondrocytes in superficial layer in OA attributed to cell clustering, another signature of OA^16^.

Given that Raman spectroscopy based technologies have been applied in arthroscopic surgeries, such as, establishing a grading system for cartilage defects^44^, this paves the way for our findings to be used in diagnostics and treatment surgery. In their work, Bergholt et al. have employed fibre-optics to quantify the ECM of living tissue constructs and applied the analysis to natural and tissue engineered cartilage^44^. Gaifulina et al.. have also reported optical probe mediated Raman for intra-operative arthroscopy utilizing the C-H and O-H stretching modes to discriminate early cartilage alterations from healthy cartilage samples^58^. With spectroscopic mapping, this approach was sensitive to reduction in GAG content and decrease in fluorescence intensity in cartilage lesions, otherwise visually indistinguishable from healthy tissue. Thus, our work could potentially be used in tandem with optical fibre probe-based and other deep-tissue interrogation methods for OA diagnosis using the spectral fingerprints or ‘biomarkers’ corresponding to different cartilage layers established in this work.

Despite being label free, minimally invasive and rapid technique with an extensive spectral data established for cartilage and bone tissue, Raman cannot provide deep tissue penetration. Recent advances in Near - infrared (NIR) and SWIR short wave infrared, non-destructive analytical techniques with deeper tissue penetration than conventional Raman spectroscopy, has proven to be sensitive to the structural and compositional changes in cartilage^64^. Although, with less resolution of the molecules detected, these can detect alterations of the extracellular matrix (ECM) providing information on the degree of cartilage degeneration in OA^65–67^. NIR-SWIR absorption spectroscopy can offer a non-destructive approach to determine thickness, biomechanical properties, and cartilage composition (water fractions) for diagnosis and assessment of OA progression^66,68,69^. Afara and colleagues have reported NIR-SWIR spectroscopic analysis of animal and human knee joint cartilage via macroscale optical fibre probes, capable of differentiating healthy vs. osteoarthritic models and track biochemical changes^65,66^. These approaches are beneficial to intraoperative or minimally invasive analysis of cartilage via a custom probe employed in surgical theatre (arthroscopy or endoscopy).

Procedures to enable early diagnosis of OA by using Raman spectroscopy with a miniaturized fibreoptic probe is minimally invasive, compared to a full surgery to replace a femoral head with an artificial one once the disease has progressed to the late stages. The early reconstructive surgery of cartilage is beneficial to prolong the lifetime of the hip joint, especially in the younger patients, but currently unavailable for OA^70^. Explorative surgery with miniaturized Raman fibreoptic probes in arthroscopy will provide “chemical” signature to the hip structures and aid in predicting OA and repairing cartilage. This procedure will be invasive and not justifiable for early stage OA diagnostics but beneficial when carried out in combination with other explorative investigations or surgeries. However, with methods such as spatially offset Raman spectroscopy^71^, signatures from deep within the tissue can be acquired and hence, it remains a possibility to use Raman spectroscopy for early diagnosis^72^.

In summary, the current work establishes the potential of OA diagnosis following examination of the Raman signatures of different cartilage layers. The current studies demonstrate that chemical changes in different cartilage layers is informative paving the way to enable early diagnosis of OA and other debilitating musculoskeletal conditions with label-free techniques such as Raman spectroscopy for an aging demographic. We have established a fingerprint of potential biomarkers for deep tissue diagnostics of OA, specifically, from the Superficial and Deep layers of the cartilage, to highlight the potential of studying differential spread of chemical, molecular and structural changes through the cartilage in disease, to show the diagnostic potential of the analysis of the whole Raman spectra rather than individual peaks with multivariant analysis. These are complemented by already available published chemical signatures of cartilage from humans, animal models as well as tissue engineered cartilage and similar connective tissues. Current findings will form the basis to seek in vivo proof–of–principle minimally invasive approaches in clinic such as a custom fibre probe for application in arthroscopy to enable early diagnosis of cartilage and matrix degenerative diseases and evaluation of implanted tissue engineered cartilage.

## Electronic Supplementary Material

Below is the link to the electronic supplementary material.

Supplementary Material 1

## Data Availability

The datasets used and analysed during the current study (Raman spectra from patients samples) available from the corresponding authors on reasonable request. The additional figures from data analysis not shown in the main part of the manuscript are included in the Supplementary section.

## Abbreviations

RS: Raman spectroscopy
CARS: Coherent anti-stokes Raman scattering
SHG: Second harmonic generation
TPH: Two photon fluorescence
OA: Osteoarthritis
PCA: Principal component analysis
PC: Principal component
LDA: Linear discriminant analysis
sGAG: Sulphated glycosaminoglycan
PCM: Pericellular matrix
