# Supplementary material for: Harnessing Raman spectroscopy and multimodal imaging of cartilage for osteoarthritis diagnosis
Source: Sci Rep. 2024 Dec 28;14:31466. doi: 10.1038/s41598-024-83155-3 (PMC11682361; doi:10.1038/s41598-024-83155-3)
Supplement: Supplementary file 1 — Supplementary Material 1 [file 41598_2024_83155_MOESM1_ESM.docx]

**Supplementary Information.**

**Table S1.** Summary of Confusion matrix scores.

| **A specific cartilage layer identified from Raman Spectroscopy data** | **Accuracy of identification in Control cartilage** | | **Accuracy of identification in OA cartilage** | |
| --- | --- | --- | --- | --- |
|  | **All spectra** | **Per patient** | **All spectra** | **Per patient** |
| **Superficial layer** | **97%** | **100%** | **92%** | **96%** |
| **Deep layer** | **93%** | **95%** | **93%** | **96%** |
| **Superficial layer (Control compared to OA)** | **71%** | **71%** | **88%** | **95%** |
| **Deep layer (Control compared to OA)** | **67%** | **84%** | **84%** | **94%** |
| **Superficial layer in under 60^th^** | **57%** | **75%** | **85%** | **88%** |
| **Deep layer in under 60^th^** | **49%** | **50%** | **96%** | **95%** |
| **Superficial layer in over 60^th^** | **58%** | **63%** | **92%** | **91%** |
| **Deep layer in OA over 60^th^** | **67%** | **77%** | **91%** | **94%** |
| **Superficial layer in Male** | **79%** | **40%** | **na** | **na** |
| **Superficial layer in Female** | **65%** | **67%** | **na** | **na** |
| **Deep layer in Male** | **82%** | **75%** | **na** | **na** |
| **Deep layer in Female** | **67%** | **73%** | **na** | **na** |

**Table S2**. Summary of main spectral peaks from measurements of superficial and deep layers in human articular cartilage and their proposed assignment.

| **Frequency (cm^-1^)** | **Assignments to chemical bonds and molecules** |
| --- | --- |
| 855 | -C-C- Proline ring (P) |
| 877 | -C-C- Hydroxyproline ring (HP) |
| 921 | -C-C- Proline ring (P) |
| 938 | -C-C- Hydroxyproline ring (HP) |
| 1004  1046 | Phenylalanine ring breathing mode of collagen and proteoglycans |
| 1033 | Phenylalanine ring breathing mode of collagen and proteoglycans, differences in collagen content |
| 1064 | SO_3_ stretching, Chondroitin sulphate (CH) (part of sGAG complex)  -C-C- skeletal saturated fatty acid |
| 1085 | -C-C- skeletal unsaturated fatty acid |
| 1101 | Hyaluronic acid (HA)  /Chondroitin sulphate (CS) /Heparan sulfate (HS) |
| 1128 | HA  -C-C- skeletal saturated fatty acid |
| 1245 | CN stretching of amide bond, Amide III, random coil (disordered) |
| 1272 | NH deformation of amide bond, Amide III α-helix or coil (ordered) |
| 1320 | CH_2_, CH_3_ twisting, Amide III, collagen twisting mode |
| 1345 | CH_2_ scissoring, Amide III, collagen bending mode. GAGS (Glycosaminoglycans) |
| 1380 | CH_3_ symmetric stretching  Lipid, GAGS (Glycosaminoglycans) |
| 1451 | CH_2_, CH_3_ scissoring, NH_2_ deformation of amide bond, collagen and other proteins |
| 1666 | NH_2_ deformation, Amide I (Collagen) |

**Table S3.** Summary of Raman peaks altered in different layers (S: Superficial, D: Deep), in OA and gender specificity.

| **(cm^-1^)** | **Assignments to chemical bonds and molecules** | **Molecules altered in S compared D layer (Controls) (1)** | **Molecules altered in S compared to D layer (OA)**  **(2)** | **Molecules altered in OA compared to control (3)** | **Molecules altered in Male compared to**  **Female (Controls)**  **(4)** | **Molecules altered in Male compared to**  **Female (OA)**  **(5)** |
| --- | --- | --- | --- | --- | --- | --- |
| 855 | -C-C- Proline ring (P) |  | V |  |  |  |
| 877 | -C-C- Hydroxyproline ring (HP) | V | V |  |  |  |
| 921 | -C-C- Proline ring (P) | V | V | V (S) | V (S) |  |
| 938 | -C-C- Hydroxyproline ring (HP) | V | V | V (S) | V (S) |  |
| 1004 | Phenylalanine ring breathing mode of collagen and proteoglycans | V |  | V (S, D) | V (S, D) | V (S, D) |
| 1064 | SO_3_ stretching, Chondroitin sulphate (CH) (part of sGAG complex)  -C-C- skeletal saturated fatty acid | V | V | V (S) | V (S) | V (S) |
| 1245 | CN stretching of amide bond, Amide III, random coil (disordered) |  | V | V (S, D) |  | V (D) |
| 1272 | NH deformation of amide bond, Amide III α-helix or coil (ordered) |  |  | V (D) |  |  |
| 1320 | CH_2_, CH_3_ twisting, Amide III, collagen twisting mode | V | V | V (S, D) | V (S) | V (S, D) |
| 1345 | CH_2_ scissoring, Amide III, collagen bending mode. GAGS (Glycosaminoglycans) | V | V | V (S, D) | V (S) | V (S, D) |
| 1380 | CH_3_ symmetric stretching  Lipid  GAGS (Glycosaminoglycans) | V | V | V (S) |  |  |
| 1451 | CH_2_, CH_3_ scissoring, NH_2_ deformation of amide bond, collagen and other proteins | V | V | V (S, D) | V (D) | V (S, D) |
| 1666 | NH_2_ deformation, Amide I (Collagen) | V | V | V (S, D) | V (S, D) | V (S, D) |

Molecules refer to chemical differences between superficial and deep layers in (1) control and (2) OA human articular cartilage samples; (3) peaks indicative of chemical changes in OA cartilage; (4) peaks indicative of chemical differences between male and female cartilage in control samples; (5) peaks indicative of chemical differences between male and female cartilage in OA samples. (S) – Superficial layer, (D) – Deep layer of cartilage, (V) indicates where changes are observed.

**
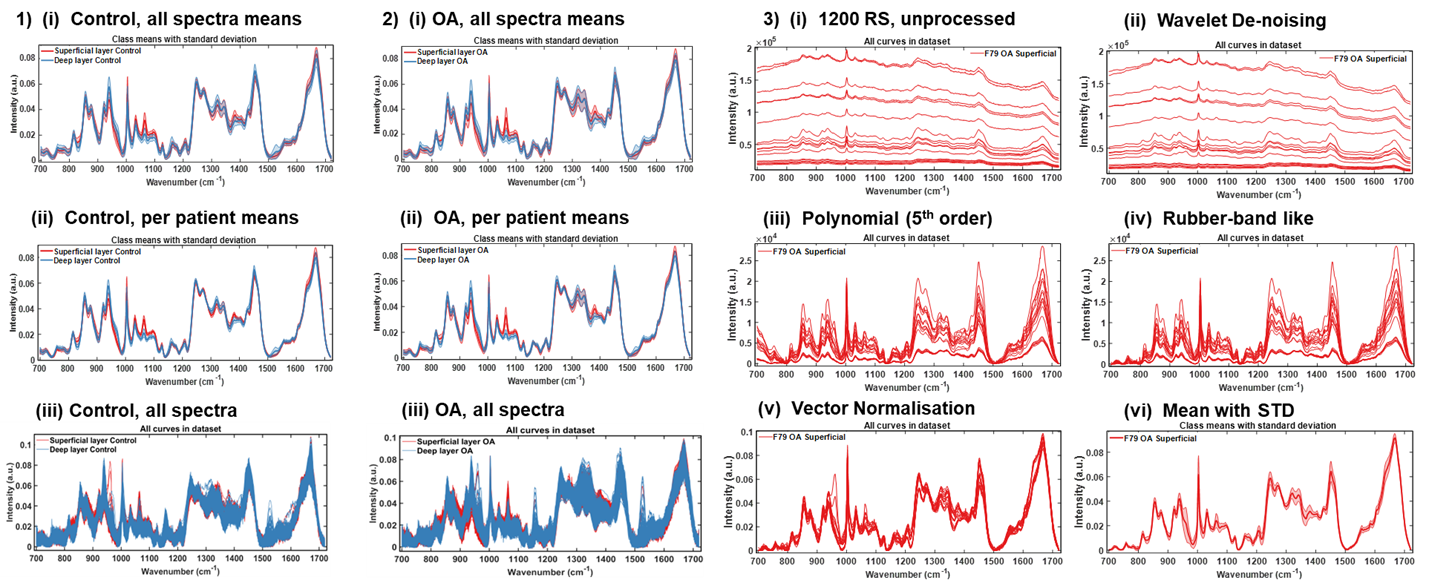
**

**Figure S1. Raman spectra of superficial and deep layers of healthy and OA cartilage.**  Fingerprint region (700 to 1720 cm^-1^) of RS of superficial (red) and deep (blue) layers of healthy “control” 1) and OA 2) cartilage. (i) Mean ± Standard Deviation of all the spectra taken from 1) Control and 2) OA cartilage. (ii) Mean ± Standard Deviation of all average/mean spectra per patient taken from 1) Control and 2) OA cartilage. (iii) All individual spectra per layer taken from 1) Control and 2) OA cartilage. Control, N=19 (male n=9, female n=10). OA, N=45 (male n=21, female n=24). 3) RS of superficial layer from F79 (OA) to demonstrate stages of processing of Raman data from each patient (i) Unprocessed data, (ii) after Wavelet De-noising, (iii) after subtracting background with 5^th^ order Polynomial, (iv) after trimming ends of spectra with Rubber-band like, (v) after normalising spectra to the arear under the curve with Vector Normalisation, (vi) processed data as Class Means with Standard Deviation. RS – Raman spectra.


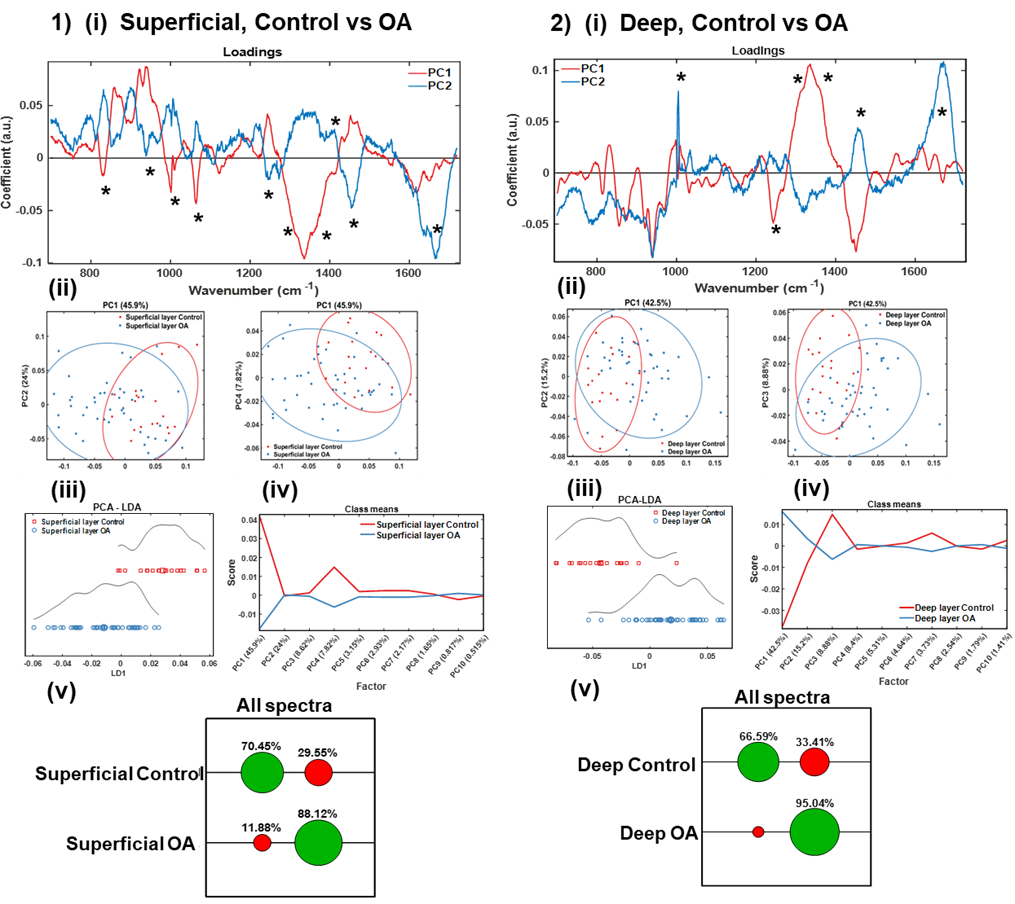


**Figure S2 (supplement to Figure 4).** Raman fingerprint for OA diagnosis of articular cartilage. 1) Superficial and 2) Deep layer (i) Loadings to demonstrate main peaks that contribute to PCA of RS spectral points in 1) (ii) and 2) (ii). Mean spectra per person are analysed in this figure, except in (v) in which all spectra for each patient are included. 1) (iii) superficial and 2) (iii) deep layer LDA analysis separates spectra into positive and negative quadrants based on classes labels from PCA analysis in (ii). 1) (iv) and 2) (iv) show class means of PC scores with percentage data variance captured by each PC. PCs with the highest score separation were included in (ii). 1) (v) and 2) (v) Confusion matrices to show accuracy of cartilage assignment to control or OA using SVM directly on the RS based on all the spectra. Green circles show positive assignment and red circles show misassignments. OA (blue) vs Control (red) cartilage.

“*” in (i) show spectral peaks contribution to PCA scores (ii), (iii). RS – Raman Spectra. PCA – Principal Component Analysis. LDA – Linear Discriminant Analysis.


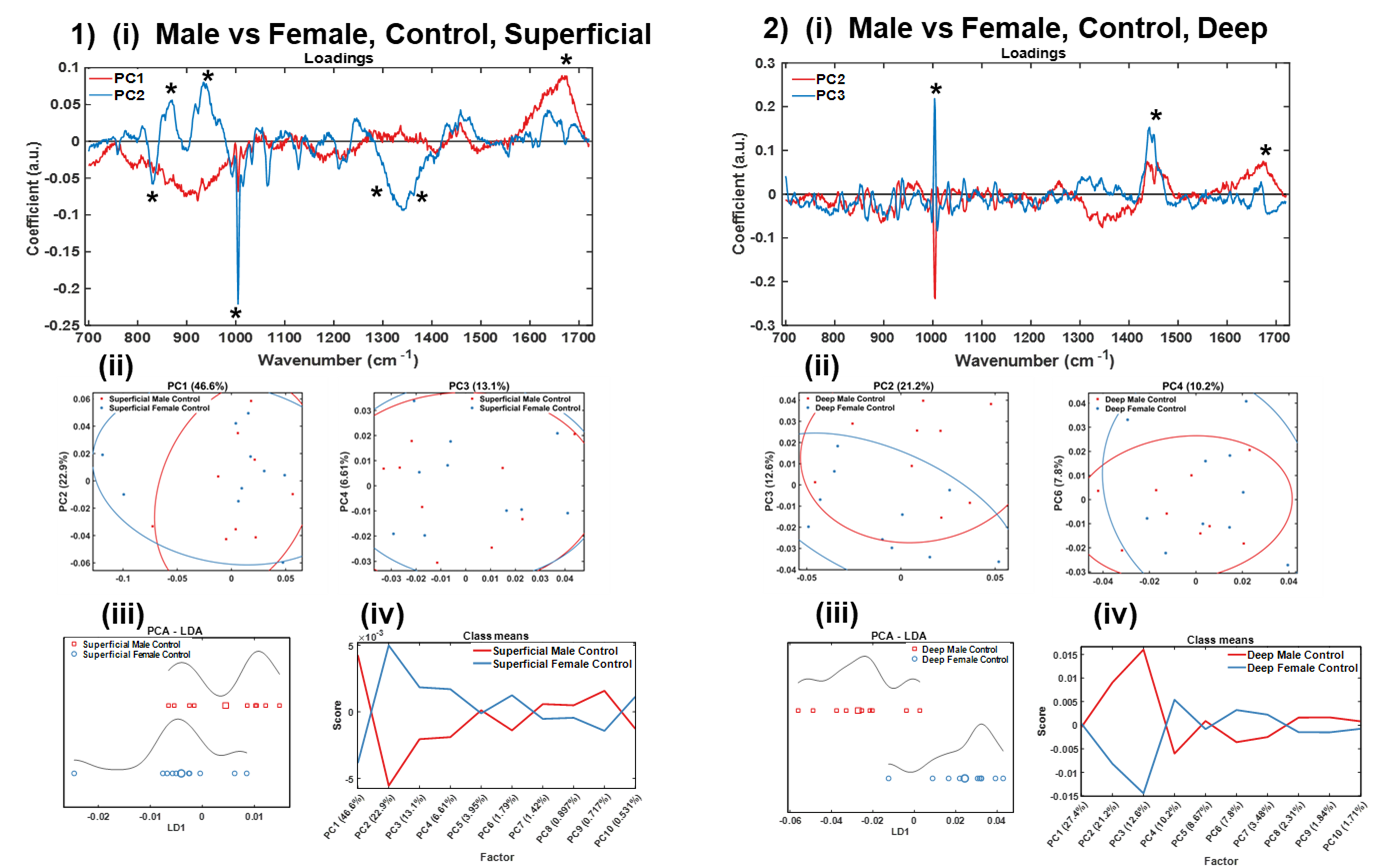


**Figure S3 (Control) (supplement to Figure 5).** Raman fingerprint for articular cartilage from gender related control samples. 1) (i) superficial and 2) (i) deep layer Loadings to demonstrate main peaks contributing to PCA of RS spectral points in 1) (ii) and 2) (ii). 1) (iii) superficial and 2) (iii) deep layer PCA - LDA analysis separates spectra into positive and negative quadrants based on classes labels from PCA analysis in (ii). 1) (iv) and 2) (iv) show class means of PC scores with percentage of data variance captured by each PC. PCs with the highest score separation were included in (ii).

Cartilage samples from Male (red) vs Female (blue) samples. “*” in (i) show spectral peaks contribution to PCA scores (ii). RS – Raman Spectra. PCA – Principal Component Analysis. LDA – Linear Discriminant Analysis.


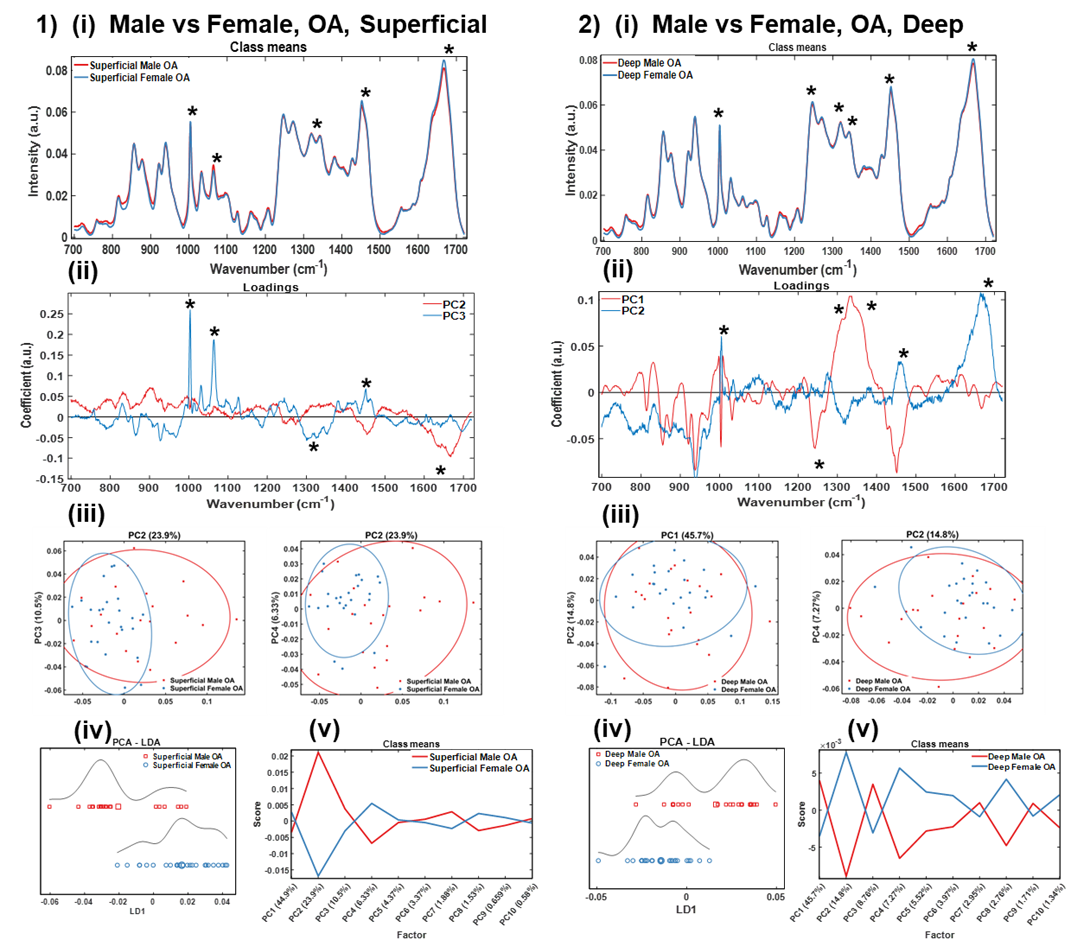


**Figure S4 (OA) (supplement to Figure 5).** Raman fingerprint for articular cartilage from gender related OA samples. 1) (ii) superficial and 2) (i) deep layer RS, mean of mean spectra per person are shown. 1) (ii) superficial and 2) (ii) deep layer Loadings to demonstrate main peaks contributing to PCA of RS spectral points in 1) (iii) and 2) (iii). 1) (iv) superficial and 2) (iv) deep layer PCA - LDA analysis separates spectra into positive and negative quadrants based on classes labels from PCA analysis in (iii). 1) (v) and 2) (v) show class means of PC scores with percentage of data variance captured by each PC. PCs with the highest score separation were included in (ii).

Cartilage samples from Male (red) vs Female (blue) samples. “*” in (i) show spectral peaks contribution to PCA scores (iii). RS – Raman Spectra. PCA – Principal Component Analysis. LDA – Linear Discriminant Analysis.

**
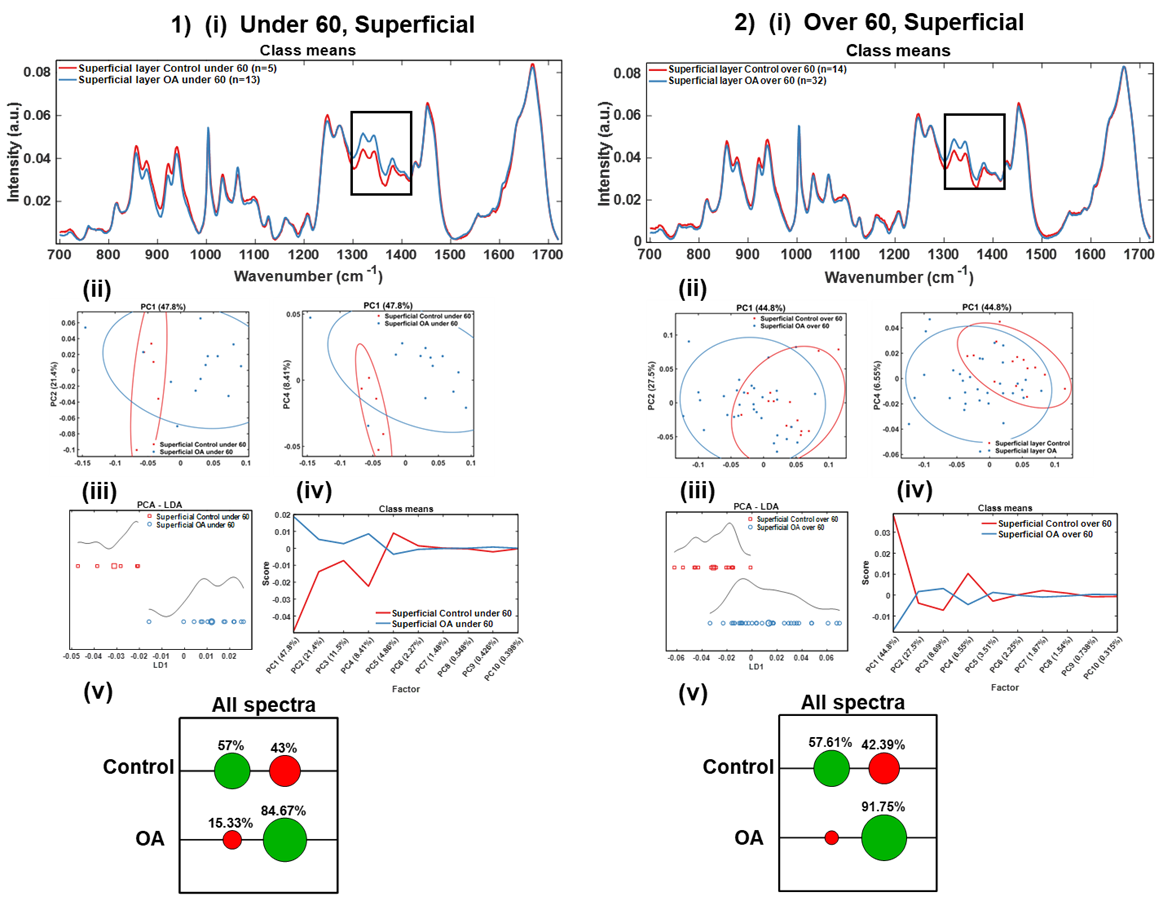
**

**Figure S5 (supplement to Figure 6).** Raman fingerprint from superficial layer of articular cartilage from age matched samples. RS of 1) (i) superficial layer, from under 60 years old (control n=5, OA n=13) and 2) (i) superficial layer, from over 60 years old (control n=14, OA n=32) individuals. Boxed part of the spectra (1320cm^-1^, 1345cm^-1^, 1380cm^-1^ peaks) show highest differences between control and OA samples. 1) (ii) and 2) (ii) 2-D representation of PCA analysis of RS spectral points from 1) (i) and 2) (i). PCA - LDA 1-D representation of 1) (iii) superficial layer, from under 60 years old and 2 (iii) superficial layer, from over 60 years old individuals separates spectra into positive and negative quadrants based on classes labels from PCA analysis in B). 1) (iv) and 2) (iv) show class means of PC scores with percentage data variance captured by each PC. PCs with the highest score separation were included in (ii). 1) (v) and 2) (v) Confusion matrices show accuracy of cartilage assignment of age matched samples to control or OA using SVM directly on the RS based on all of the spectra. Green circles show positive assignment and red circles show misassignments. OA (blue) vs Control (red) cartilage.

Control (red) vs OA (blue) samples. RS – Raman Spectra. PCA – Principal Component Analysis. LDA – Linear Discriminant Analysis.


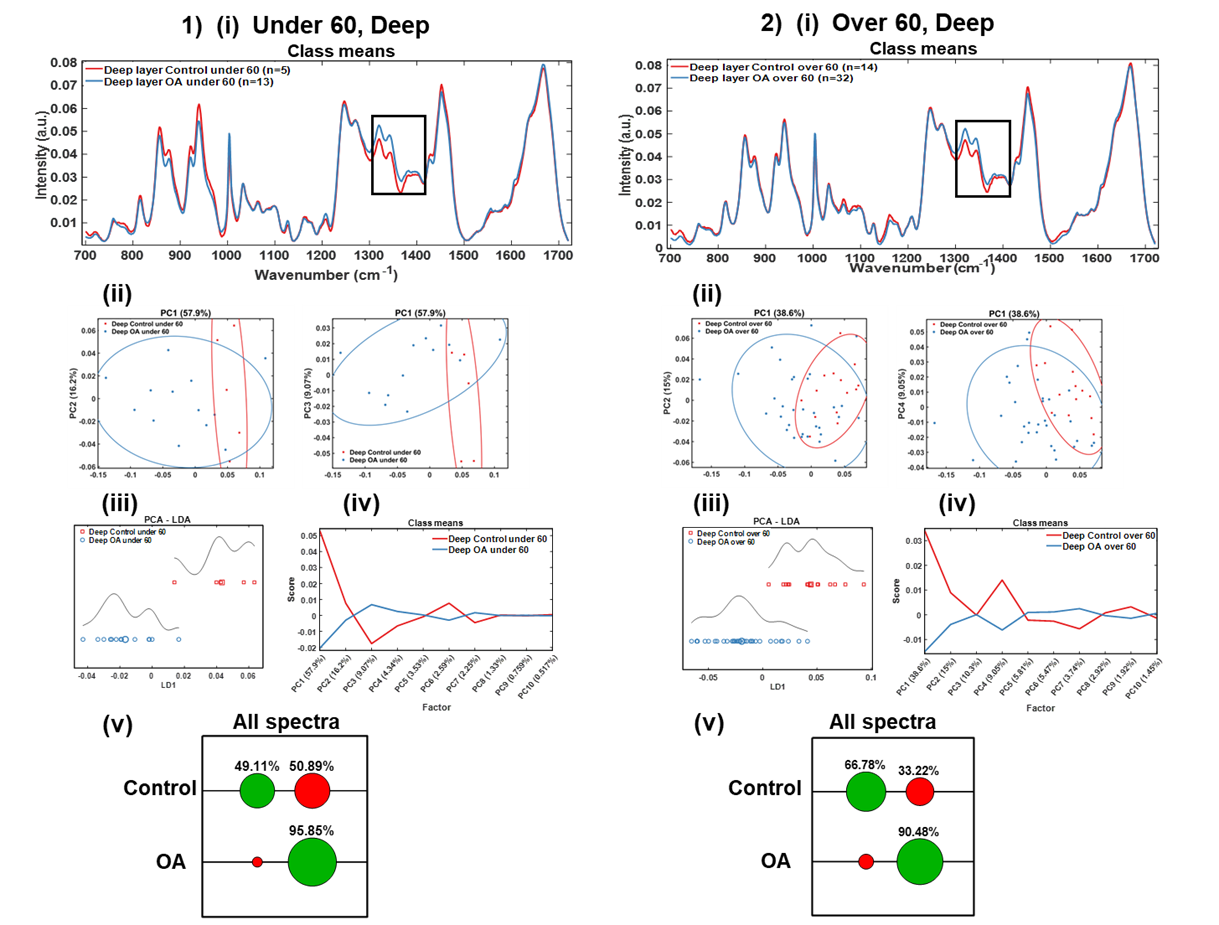


**Figure S6 (supplement to Figure 6).** Raman fingerprint from deep layer of articular cartilage from age matched samples. RS of 1) deep layer, from under 60 years old (control n=5, OA n=13) and 2) deep layer, from over 60 years old (control n=14, OA n=32) individuals. Boxed region of the spectra (1320cm^-1^, 1345cm^-1^, 1380cm^-1^ peaks) show highest differences between control and OA samples. 1) (ii) and 2 (ii) 2-D representation of PCA analysis of RS spectral points from 1) (i) and 2) (i). PCA - LDA 1-D representation of 1) (iii) deep layer, from under 60 years old and 2 (iii) deep layer, from over 60 years old individuals separates spectra into positive and negative quadrants based on classes labels from PCA analysis in (ii). 1) (iv) and 2) (iv) show class means of PC scores with percentage data variance captured by each PC. PCs with the highest score separation were included in (ii). 1) (v) and 2) (v) Confusion matrices to show accuracy of cartilage assignment of age matched samples to control or OA using SVM directly on the RS based on all the spectra. Green circles show positive assignment and red circles show misassignments.

Control (red) vs OA (blue) samples. RS – Raman Spectra. PCA – Principal Component Analysis. LDA – Linear Discriminant Analysis.

**
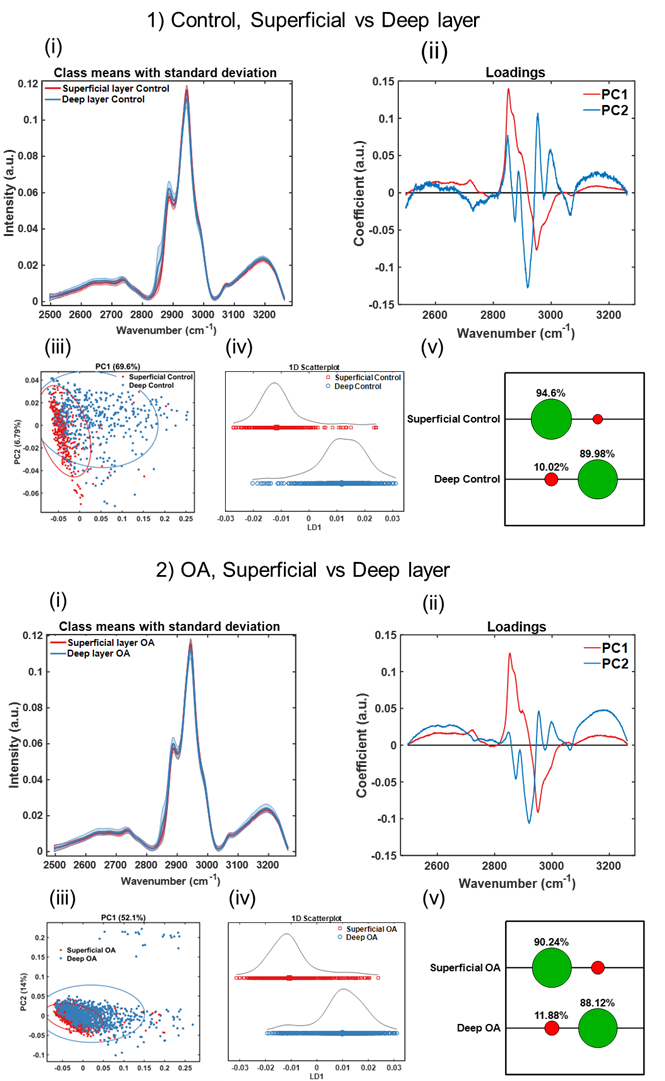
**

**Figure S7.** Raman spectra at CH_2_/CH_3_ region for comparison of superficial and deep layers of articular cartilage. Top panel 1) control samples (n=19), bottom panel 2) OA samples (n=45). 1) and 2) (i) RS of CH region (2500 to 3300) of superficial (red) and deep (blue) layers of cartilage, Mean ± STD. 1) and 2) (ii) Loadings from PC1 and PC2 PCA scores in (iii). 1) and 2) (iii) 2-D scatter plot demonstrating distribution of superficial and deep layer spectra along PC1 and PC2 axes. 1) and 2) (iv) LDA analysis shows further separation of superficial (red) and deep (blue) layers based on class labels from PCA. 1) and 2) (v) Confusion matrices based on SVM analysis of all spectra per patient. Control samples: mean age 72 years, median age 78 years, n=19 (male=9, female=10). OA samples: mean age 69 years, median 73 years, n=45 (male=21, female=24).


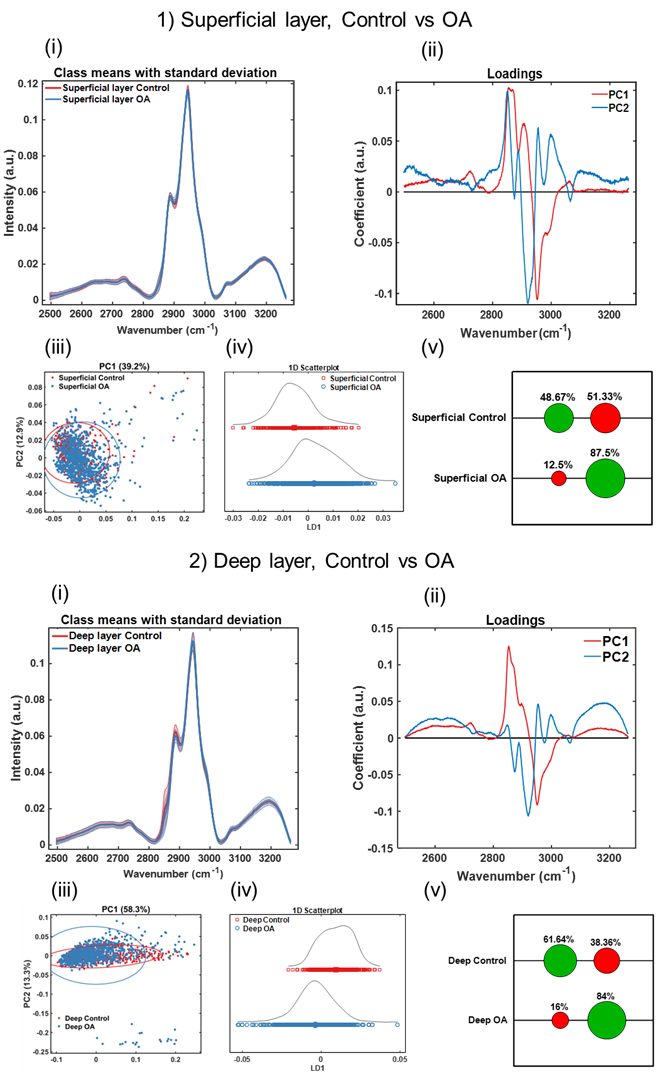


**Figure S8.** Raman spectra at CH_2_/CH_3_ region for OA diagnostics of articular cartilage. 1) and 2) (i) RS of CH region (2500 to 3300) of control (red, n=19) vs OA (blue, n=45) samples; superficial layer in top panel 1), deep layer in bottom panel 2), Mean ± STD. 1) and 2) (ii) Loadings from PC1 and PC2 PCA scores in 1) and 2) (iii). 1) and 2) (iii) 2-D scatter plot demonstrating distribution of superficial and deep layer spectra from Control vs OA cartilage along PC1/PC2 axes. 1) and 2) (iv) 1-D LDA analysis of superficial 1) (iv) and deep 2) (iv) control (red) and deep (blue) layers based on class labels. (v) Confusion matrices based on SVM analysis of all spectra per patient for Superficial layer 1) (v) and Deep layer 2) (v). Control samples: mean age 72 years, median age 78 years, n=19 (male=9, female=10). OA samples: mean age 69 years, median 73 years, n=45 (male=21, female=24).
